# A unified compendium of prokaryotic and viral genomes from over 300 anaerobic digestion microbiomes

**DOI:** 10.1186/s40793-023-00545-2

**Published:** 2024-01-02

**Authors:** Victor Borin Centurion, Alessandro Rossi, Esteban Orellana, Gabriele Ghiotto, Balázs Kakuk, Maria Silvia Morlino, Arianna Basile, Guido Zampieri, Laura Treu, Stefano Campanaro

**Affiliations:** 1 Department of Biology, University of Padua, Via U. Bassi 58/B, 35131 Padua, Italy; 2 Department of Medical Biology, Albert Szent-Györgyi Medical School, University of Szeged, 12 Somogyi B. U. 4., Szeged, 6720 Hungary; 3 MRC Toxicology Unit, University of Cambridge, Gleeson Building Tennis Court Road, Cambridge, UK

**Keywords:** Anaerobic digestion, Metagenomics, Bacteriophage, Biogas, Functional annotation, Microbe, Metagenome-assembled genome

## Abstract

**Background:**

The anaerobic digestion process degrades organic matter into simpler compounds and occurs in strictly anaerobic and microaerophilic environments. The process is carried out by a diverse community of microorganisms where each species has a unique role and it has relevant biotechnological applications since it is used for biogas production. Some aspects of the microbiome, including its interaction with phages, remains still unclear: a better comprehension of the community composition and role of each species is crucial for a cured understanding of the carbon cycle in anaerobic systems and improving biogas production.

**Results:**

The primary objective of this study was to expand our understanding on the anaerobic digestion microbiome by jointly analyzing its prokaryotic and viral components. By integrating 192 additional datasets into a previous metagenomic database, the binning process generated 11,831 metagenome-assembled genomes from 314 metagenome samples published between 2014 and 2022, belonging to 4,568 non-redundant species based on ANI calculation and quality verification. CRISPR analysis on these genomes identified 76 archaeal genomes with active phage interactions. Moreover, single-nucleotide variants further pointed to archaea as the most critical members of the community. Among the MAGs, two methanogenic archaea, *Methanothrix* sp. 43zhSC_152 and *Methanoculleus* sp. 52maCN_3230, had the highest number of SNVs, with the latter having almost double the density of most other MAGs.

**Conclusions:**

This study offers a more comprehensive understanding of microbial community structures that thrive at different temperatures. The findings revealed that the fraction of archaeal species characterized at the genome level and reported in public databases is higher than that of bacteria, although still quite limited. The identification of shared spacers between phages and microbes implies a history of phage-bacterial interactions, and specifically lysogenic infections. A significant number of SNVs were identified, primarily comprising synonymous and nonsynonymous variants. Together, the findings indicate that methanogenic archaea are subject to intense selective pressure and suggest that genomic variants play a critical role in the anaerobic digestion process. Overall, this study provides a more balanced and diverse representation of the anaerobic digestion microbiota in terms of geographic location, temperature range and feedstock utilization.

**Supplementary Information:**

The online version contains supplementary material available at 10.1186/s40793-023-00545-2.

## Introduction

The increasing demand for energy and the depletion of fossil fuels have shifted attention towards alternative energy production processes. One such process is anaerobic digestion (AD), which has a lower environmental impact, while supporting the concept of circular economy by utilizing a variety of end-products including food, agricultural, industrial and municipal wastes. AD is a natural process that breaks down complex organic matter into simpler compounds, occurring in ecological niches with low oxygen or strictly anaerobic conditions, such as bogs, sediments and the guts of herbivores [1]. This process is also utilised at industrial-scale activities for biogas production. The breakdown is carried out by a microbial community that can range in complexity from a few species to an extremely complex microbiome consisting of thousands of species [2–4]. The biogas obtained from industrial reactors typically contains a mixture of methane (CH_4_) and carbon dioxide (CO_2_), with trace levels of hydrogen sulfide (H_2_S), ammonia (NH_4_^+^), hydrogen (H_2_), and various volatile organic compounds, depending on the feedstock and on the functional activity of the microbiota [5].

From a biotechnological perspective, CH_4_ is the most significant constituent of the biogas generated during the methanogenesis step of the AD process and is produced by methanogenic Archaea [6, 7]. AD is carried out by a diverse community of microorganisms, where each species has a unique role in highly specialized and complex microbiomes [8]. First, complex organic matter is transformed by hydrolytic bacteria into soluble organic compounds. Second, acidogenic bacteria transform the latter into intermediates (e.g., volatile fatty acids), with syntrophic acetogenesis playing an essential role in breaking compounds down into the simplest molecules. Third, methanogenesis is performed by archaea using acetate, methylamine and/or CO_2_ and H_2_ to produce CH_4_. Understanding the composition and role of each species in the community is crucial for a better understanding of the carbon cycle in anaerobic systems and improving biogas production. However, the isolation of many species using classical microbiological techniques can be challenging, making metagenomics an ideal alternative for characterizing the community complexity. In addition to the microbiome, the efficiency of AD is dependent on several interconnected factors, including feedstock composition, temperature, organic loading rate, hydraulic retention time, and other physicochemical parameters [9, 10]. Controlling these parameters can offer unique opportunities for microbial selection or manipulation to improve the process efficiency.

The rise of metagenomics in the last two decades has revealed the important role viruses play in shaping microbial communities [11]. In many environments viruses are responsible for selective pressure, lateral gene transfer, and nutrients recycling, all of which impact the microbiota. Understanding their role at community level is crucial and offers an opportunity to fine-tune the AD process. Despite the important role viruses play, the viral community of AD has received little attention [12, 13]. This gap is due to difficulties associated with viral metagenomics compared to the prokaryote investigation. To separate the viral fraction of the community from the microbial is challenging, as viruses represent a small fraction of the total genetic material. Therefore, viruses are more understudied than bacteria and archaea. However, research has shown that phages, in particular, are involved in microbiome dynamics and process stability, regulating microbial abundance and diversity in full-scale biogas units [14].

Metagenome sequencing has become a valuable tool to gain insights into the genetic repertoire of non-cultivable biogas community members [15]. Advances in sequencing throughput and computational techniques nowadays allow the recovery of Metagenome-Assembled Genomes (MAGs) from highly diverse environments [16]. These MAGs are obtained through binning together assembled contigs with similar sequence composition, depth of coverage, and taxonomic affiliations [17–19]. Despite limitations regarding completeness and contamination [20], MAGs are useful proxies for studying microbial and viral genomes present in the system, providing insights into taxonomy, functional properties, and dynamics of the microbiome. Several studies have attempted to gain insights into the AD microbiome for biogas production [15, 21], but the limited geographic distribution of the samples and the lack of a global analysis on the viral fraction have prevented a complete characterization of the microbiome. To address this gap, a global meta-analysis study was performed to tentatively assess the impact of physicochemical parameters, microbiota, and reactor characteristics on the AD process. The aim of this study was to complement and consolidate previous results and establish a more comprehensive reference database of microbial and viral genomes. In addition, strain-resolved metagenomics was applied to reveal fine-scale evolutionary mechanisms, functional dynamics, and strain-level metabolic variation, potentially contributing to the selection within a microbial community.

## Materials and methods

### Collection of samples and metadata

In order to expand the Biogas Microbiome database [15], 192 additional metagenomic datasets were retrieved from SRA using the SRA toolkit fastq-dump software v2.10.8 [22]. The present version of the database now includes details regarding the 314 metagenomes derived from AD biogas reactors, which were identified through searches on SRA and the literature (Additional file 1). Only datasets published between 2014 and 2022 were considered, with metadata taken from the respective experiments when available. Temperature information was present in most experiments and classified as psychrophilic (0–19 °C), mesophilic (20–40 °C) and thermophilic (41–56 °C). The metadata for the additional datasets can be checked in Additional file 1. Reads were filtered and quality checked as previously described [23] using a two-step procedure: at first Trimmomatic v0.39 [24] was used in its paired-end mode, using the parameters leading = 20, trailing = 20, slidingwindow = 4:20 and minlen = 70, at second, a further check was performed to remove adapters and contaminants associated to phiX174 with BBDuk v38.86 [25]; filtering was performed using the files “adapters.fa” and “phix174_ill.ref.fa” as references, and the parameters k = 21 ktrim = r mink = 11 hdist = 2 threads = 4.

### Assembly, binning, and coverage

Metagenome assembly was performed independently for all the samples of one experiment using MEGAHIT v1.2.9 [26]. Assembly statistics were obtained using QUAST v5.0.2 [27]. Bowtie2 [28] was used to map reads back to the contigs for the creation of coverage files for the binning analysis. Every assembly was binned both with MetaBAT2 v2.12.1 [29] and MaxBin v2.2.7 [30] using standard parameters. Previous experiments, which underwent analysis using MetaBAT2 only, were binned with MaxBin. 11,781 MAGs previously generated by Ma and colleagues [21] were downloaded from “http://dx.doi.org/10.5524/100842” and included in the database. Completeness and contamination of MAGs generated in this study and in MAGs generated by Ma and colleagues were assessed with CheckM v1.1.2 [31] and CheckM2 v0.1.2 [32] for comparative assessments across different versions of the software. MAGs with completeness lower than 50% and contamination higher than 10% were filtered out. The two sets of medium–high quality MAGs were combined and used as input for the dereplication step. MAGs were dereplicated with dRep v3.2.2 [33] using parameters coherent with the previously proposed definition of species [34]: 95% average nucleotide identity on at least 50% of the MAG genome. To ensure sequencing depth homogeneity, libraries were subsampled to 8 million reads and mapped onto the MAGs with Bowtie2 v2.5.0 [28]. For each prokaryotic MAG, the relative abundance and reads counts were calculated with coverM (https://github.com/wwood/CoverM) using the “genome” subcommand. Alpha-diversity was calculated with the “estimate_richness” function of Phyloseq v1.40.0 R package [35] using the mapped reads counts on the MAGs. Beta-diversity was calculated with ExpressBetaDiversity v1.0.10 [36] using Bray–Curtis dissimilarity and the relative abundances of prokaryotic MAGs. Virus predicting software PPR-Meta v1.1 [37], CheckV v0.7.0 (end_to_end program) [38], and VIBRANT v1.2.0 [39] were launched on the assembled contigs. A contig was assigned as viral if the prediction was independently confirmed by at least two of the applied programs. All the scaffolds predicted as viral but shorter than 5 kbp were removed to reduce the number of mispredicted and partially assembled viral genomes.

### Taxonomy and functional prediction

GTDB-Tk v1.4.1 [40] was used to taxonomically assign prokaryotic MAGs, with the release 95 of Genome Taxonomy Database (GTDB) serving as reference database. Prodigal v2.6.3 [41] was used to predict protein-encoding genes, and functional annotation was carried out with eggnog-mapper v2.1.7 [42] on dereplicated MAGs and viral genomes. The completeness of KEGG modules was determined for each MAG using KEMET (release version July 2022) [43] with the parameter '-a eggnog' utilized to incorporate the eggNOG annotations. MAG functions were assessed by manually inspecting specific KEGG modules related to functional classes of AD: methanogenesis (M00357, M00567); beta-oxidation (M00087); anaerobic carbon metabolism (M00173, M00377, M00618); nitrogen metabolism (M00528, M00529, M00530, M00531, M00615, M00804); sulfate reduction (M00176, M00596). For phylogenetic analysis of MAGs, PhyloPhlAn 3.0.51 [44] was used with the parameters—diversity high and—fast, and PhyloPhlAn database. The resulting Newick file was visualized using iTOL v6.7 [45]. Taxonomic assignment of phages was carried out using PhaGCN v2.0 [46] considering contigs above 5kbp (–len 5000).

### Replication rate estimation

Species replication rates across the 8 million reads subsamples were estimated using CoPTR v1.1.4 with default parameters [47]. This approach estimates the slope of read coverage on genomes or MAGs assuming that fast-replicating microbes are associated with increasing DNA abundance the closer a sequence is to the origin of replication. Such a slope is named peak-to-trough ratio (PTR) and quantifies DNA synthesis and generation rate in terms of an adimensional coverage decay rate, which in turn is a proxy of microbial replication rate. To guarantee robust PTR estimates, MAGs with minimum 5,000 aligned reads were selected for this analysis.

### CRISPR detection

MinCED v0.4.2 [48] was used to identify clustered regularly interspaced short palindromic repeats (CRISPR) spacers in the dereplicated MAGs. A BLAST v2.6.0 [49] search for viral sequences recovered in this study against the spacers was performed using parameters including -task blastn-short, -gapopen 10, -gapextend 2, -penalty -1, -word_size 7, -perc_identity 100, as previously reported [50]. The results were filtered to include only those that matched the whole length of the spacer. To visualize interactions between Phages and Archaea, HoloViews v1.15.4 (https://github.com/holoviz/holoviews), along with Pandas (v2.3.1) and NumPy (v1.21) packages in python v3.6.13 were used. The phage coverage was estimated using CoverM v0.6.1 with the “contig” subcommand. To compare the presence of phages in different experiments for each virMAG, the abundance was divided by the total number of reads in each sample.

### Strain analysis

The analysis of variants was performed using the tool InStrain v1.6.3 [51], specifically the module “profile”. To do this, a scaffold-to-bin-file establishing correspondences between contigs and 160 MAGs was generated using the parse_stb.py script from the dRep v3.2.2, and the ORF prediction file obtained using Prodigal v2.6.3. InStrain profile was launched with additional parameters: “–min_genome_coverage 1”, “–skip_plot_generation”, “–min_mapq 2”, “–min_read_ani 0.98” and “–skip_mm_profiling”. A MAG was deemed present in the sample only if it exhibited a breadth of coverage greater than 0.5. Variants were then filtered based on the procedure outlined in Ghiotto et al. (2023) [52]. The Mann–Whitney U-test was employed to evaluate the accumulation of variants within a specific phylum in comparison to the overall population. The distribution of SNVs/Mbp for each phylum was compared against the SNVs/Mbp of all MAGs containing SNVs.

### Statistical analysis

The Pearson correlation between Chao1, Shannon alpha-diversity indices, temperature, and checkM and checkM2 quality estimates was calculated in R v4.2.1 utilizing ggpubr v0.6.0. The cumulative abundance of archaeal MAGs was defined as the sum of all samples relative abundance with complete and one block missing of M00357 (Methanogenesis, acetate =  > methane) and M00567 (Methanogenesis, CO2 =  > methane) KEGG modules. The read counts of MAGs with complete and one block missing M00357 and M00567 KEGG modules were filtered and values normalized as relative abundance. The data were then plotted using the ComplexHeatmap v2.12.1 R package [53].

To determine the relationship between PTR and process temperature for individual MAGs, those detected in at least five different temperature values spanning a minimum range of 10 °C were selected, in order to consider a reasonable temperature spectrum. For the selected MAGs, second-order ordinary polynomial models were fitted with temperature and PTR as independent and dependent variables, respectively, using the lm function in R v3.6. To correct for multiple testing, the *p*-values were adjusted for linear and quadratic coefficients using the Benjamini–Hochberg method. Fits that had at least one significant coefficient at a 0.1 FDR level were selected. Finally, preferential temperature ranges were calculated by considering PTR values greater or equal to the median PTR of each MAG independently, and then taking maximum and minimum temperatures for the conditions where these were detected. Only MAGs having at least three PTR values available were considered in this case.

## Results and discussion

### Expansion of the global anaerobic digestion microbiome database

**Fig. 1:**
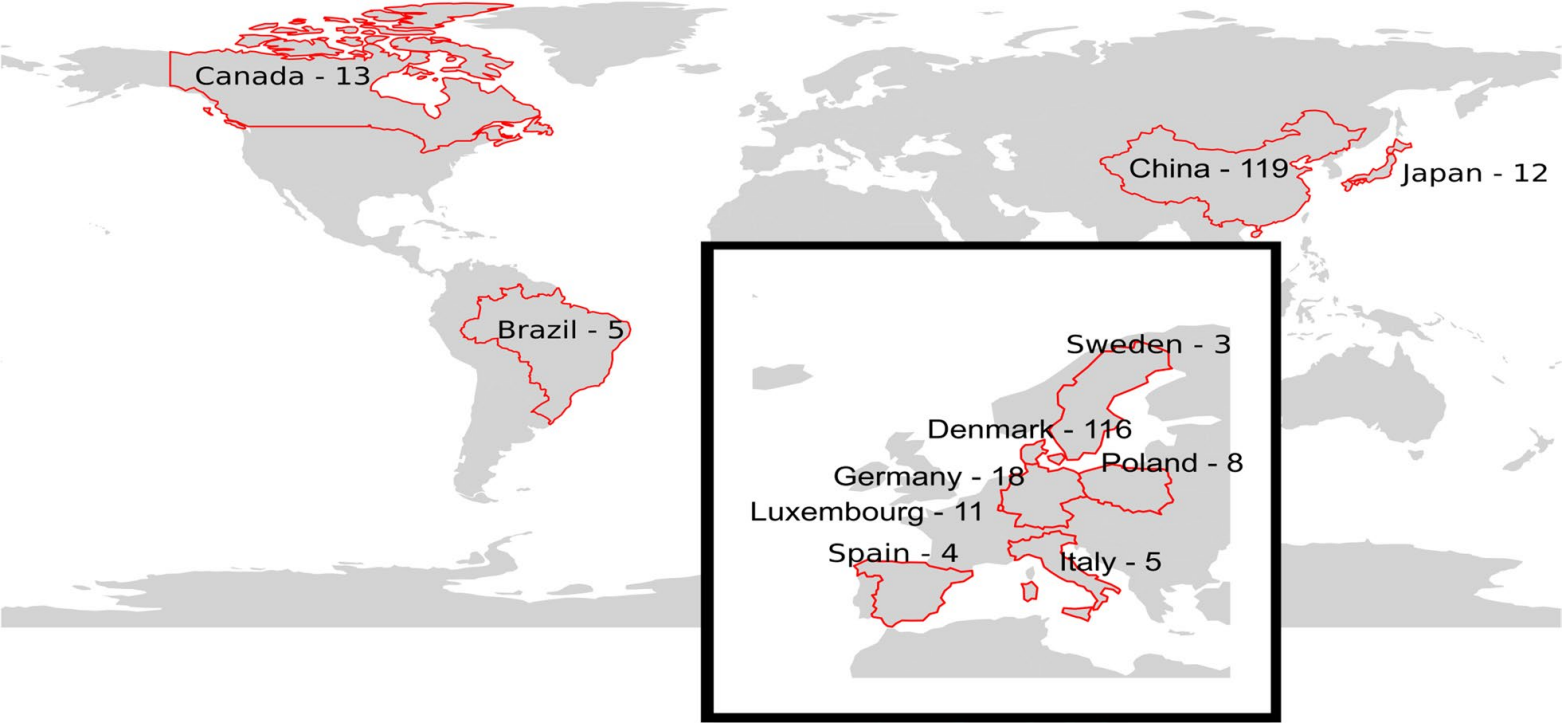
Geographic and microbial diversity of the expanded Biogas Microbiome database. The expanded anaerobic digestion microbiome database includes 314 samples distributed across 11 countries, with Denmark and China being the origin of most samples. Information about all the samples can be visualized in Additional file 1

To create a more balanced representation of MAGs from various countries, the previous iteration of the Biogas Microbiome database featured sequences from 18 studies, containing 123 samples [15]. However, it was biased towards samples collected from Denmark, which accounted for 68% of the database, mostly deriving from laboratory-scale biogas reactors and batch tests. In the present version, we added 24 studies to the database, resulting in a total of 314 samples. Notably, a significant number of samples and MAGs, derived from Chinese biogas plants, were included in a recent study and implemented in the new database [21]. This expanded Biogas Microbiome database now encompasses samples collected in eleven countries (Fig. 1, Additional file 1), with China and Denmark being the primary contributors, accounting for 75% of the total number of samples. Samples were categorized according to reactor type, with 111 described as “lab scale” or “batches” operated in six countries, 104 described as “full-scale biogas plants”, and 99 as “(semi)continuous lab reactors” or “CSTR” (Additional file 1).

To gather data, only basic statistics were collected from the metadata of different publications. The data were evenly distributed across different temperatures, with 4% being psychrophilic, 51% mesophilic, and 38% thermophilic; 22 samples had no clearly reported operating temperature or reactor type. This study provides a more comprehensive representation of microbiota structure and species growing over a wider range of temperatures, compared to previous investigations [15]. Temperature is an essential factor in determining the diversity of the community, as it was found to be inversely correlated with Chao1 and Shannon alpha-diversity indices (Pearson’s r = − 0.4, *p* = 1·10^–12^) (Additional file 2: Fig. 1). The three samples with the highest diversity were collected from reactors operated at the lowest temperature, such as Ma_2021_BGP_27, 29, and 34, with temperatures ranging from 14 to 17 °C and having Chao1 ∼4,566 and Shannon ∼6.37 (Fig. 1). Previous studies have reported that psychrophilic temperatures have more diversity of methanogenic Archaea [54] and fluctuation in rare biosphere taxa [55] than mesophilic reactors, which represented the majority of the samples in this study. However, psychrophilic temperatures generally slow down the metabolic activity of anaerobic microorganisms. This can result in a prolonged retention time for organic matter in the digester and lead to a decrease in methane yield per unit of organic matter compared to mesophilic or thermophilic conditions [56, 57].

### Prokaryotic community composition

The binning process generated 11,831 MAGs, which, when combined with the database by Ma and colleagues [21], resulted in a total of 26,612 MAGs. To reduce redundancy, 4,568 MAGs were selected for clustering based on ANI calculation and quality analysis with CheckM. Of these, 2,217 (48.5%) were classified as high quality (completeness >  = 90%; contamination <  = 10%) while 2,351 (51.5%) were of medium quality (90% > completeness >  = 50%; contamination <  = 10%) (see Availability of Data and Materials). These results were validated with CheckM2 and presented a high correlation with those of CheckM (R^2^ = 0.7). This concordance is especially relevant as it demonstrates the accuracy of CheckM2 on a large dataset, while still validating quality assessments obtained with CheckM. Specifically, the estimated completeness was higher for 59% of the MAGs, lower for 40.8%, and 0.24% showed the same value as CheckM. Notably, out of the 387 *Candidatus* MAGs, 293 (75.7%) had higher completeness scores with CheckM2, and 57 (14.7%) had completeness scores that were two times higher than CheckM. Additionally, 76% of the samples had more than 50% mapped reads, indicating that this version of the Biogas Microbiome database predominantly characterizes the microbiome. The batch experiment enriched culture with cellulose (Jia_2018_Batch_12 and 13) [58] had the highest proportion of mapped reads, with about 94% of bacterial MAGs mapped.

Taxonomic investigation on the prokaryotic community revealed that members of Firmicutes dominate, comprising 38% of the MAGs in the database, followed by Proteobacteria (12%) and Bacteroidetes (10%). Archaea are represented by 198 species (4.3%), mainly from the Euryarchaeota phylum (76.3%), while Candidatus Bathyarchaeota and Candidatus Diapherotrites are represented by 13 and 12 members, respectively. Previous metataxonomy-driven investigations [15] identified 53 MAGs belonging to the Euryarchaeota phylum while the other Candidatus phyla were not represented. Moreover, among the archeal population, Euryarchaeota were similarly identified as the predominant phylum in another large-scale study examining the microbiome of 80 anaerobic digesters [59]. Among all the MAGs identified, only a small number (3.7%) were assigned at species level (152 Bacteria and 19 Archaea), while 19.3% were assigned at genus level (794 Bacteria; 87 Archaea). These results confirm that the fraction of archaeal species already characterized at genome level and reported in public repositories of microbial genomes is higher (9.6%) than that of bacteria (3.5%), but still very low.

Analysis performed on MAG relative abundance in all the samples provided insight into the distribution of microbial species in the database. By calculating the number of samples in which each MAG abundance was greater than 0.01, 0.1, and 1%, Firmicutes, Euryarchaeota, Bacteroidetes, Proteobacteria, and Synergistetes were identified to be more widespread, while others, such as Fibrobacteres, Ignavibacteriae, Crenarchaeota, and many *Candidatus* phyla had a more scattered distribution (Additional file 2: Fig. 2). Similar results were obtained using 0.1% or 0.01% as the relative abundance threshold to define the presence of a MAG (see Availability of Data and Materials). Strikingly, all the four bacterial strains enriched in previously characterized biofilm communities developed on the gas injection systems of biogas upgrading reactors were among the top widespread MAGs across samples at all relative abundance thresholds [60]. These MAGs include Firmicutes 50dbBF_058, Firmicutes 50dbBF_049, Firmicutes 50dbBF_057, and Synergistaceae sp. 24abBP_148. In particular, the most widely detected MAG Firmicutes 50dbBF_058 is alternatively classified as Limnochordia DTU010 and was previously found to possess the glycine cleavage system and the glycine synthase-reductase pathway for CO_2_ reduction, which are considered important for establishing syntrophic interactions with methanogens [60]. Other 45 less widespread MAGs were here assigned to Limnochordia DTU010 and several species of the same order have also recently been identified across a numerous independent set of full-scale plants in sensible abundance [61], further raising our interest in this uncharacterized taxon. Besides, MAGs belonging to the phylum *Candidatus* Atribacteria were widespread but with a low relative abundance (less than 0.1%). This finding suggests that methanogenic Archaea and Synergistetes, for which only a small number of MAGs have been identified in the database, play crucial roles and are very flexible, being able to adapt to a variety of environmental conditions. The two most widespread Archaea, *Methanothrix sp.* 43zhSC_152 and *Candidatus Methanoculleus thermohydrogenotrophicum* 31mySI_10, were detected in 49 and 42 samples, respectively, with relative abundance ≥ 1%. Similarly, the two most common Synergistetes MAGs, Synergistaceae sp. 24abBP_148 and *Acetomicrobium flavidum* 43zhSC_162, were present in 35 and 15 samples at relative abundance ≥ 1%. In fact, members of the Synergistaceae taxon were previously found to show acetate-oxidizing ability, which may work in syntrophy with hydrogenotrophic methanogens for methane production [62, 63]. This crucial cooperation activity may explain the similar common widespread distribution of these two phyla. On the other hand, some Candidate phyla and Planctomycetes have a scattered distribution. Genome size is correlated with the number of genes and, on average, Planctomycetes have the second-highest genome size. This suggests that the ability to colonize many different samples is not necessarily related to species gene content. The highest archaea/bacteria ratio was found in a thermophilic batch reactor fed with a synthetic medium containing methanol (Yan_2020_Batch_2, Additional file 1). The Archaea in Yan and colleagues [62] is represented by one MAG from the Euryarchaeota phylum with 100% completeness which could not be taxonomically assigned with higher specificity, indicating the need for a more detailed taxonomic investigation on some Archaea branches.

### Phage-prokaryote interactions indicate shared adaptation strategies

A total of 79,922 viral genomes were identified after dereplication, and 32% of them were successfully classified. The composition of the viral community was largely dominated by tailed bacteriophages of the class *Caudoviricetes*, as established by previous works [12, 13, 64, 65], comprising 97.6% of all viruses. Families *Straboviridae*, *Peduoviridae*, and *Herelleviridae* were the most represented in the dataset, with 2,927, 2,887, and 2,825 members, respectively. However, the family with the greatest coverage in the 268 samples was *Peduoviridae*, followed by *Straboviridae* and *Drexlerviridae* (Additional file 2: Fig. 3B, see Availability of Data and Materials for full statistics). The virome of the 314 samples analyzed represented between 8 × 10^–4^ and 0.86% of the total community. As expected, 11 samples from an enrichment of viromes experiment [12] had a higher proportion of phages (0.35–0.86%). However, the virus with the highest proportion, virMAG MPIJXP02F1_92297, was found at low abundance in these samples, while thermophilic reactors fed with exhausted gasses and carbohydrates had a higher abundance of this virus. Although PhaGCN was not able to taxonomically identify this phage, the presence of enzymes related to DNA binding domains (pfam13443), transposase related to bacteriophages (COG5421), and DNA-binding transcriptional regulator (COG3655) confirmed its classification as a viral genome. Permanova analysis revealed a significant correlation (*p* < 0.01) between the temperature of the reactor and the presence of different phages (Additional file 2: Fig. 3A). Considering taxonomically classified phages with relative abundance over 0.3%, 27 out of 47 families were influenced by temperature. *Rudiviridae* predominated in reactors below 25 degrees, while *Suoliviridae* (n = 527) and *Winoviridae* (n = 256) were most represented among the 10 dominant families in mesophilic environments. In thermophilic environments, *Peduoviridae*, *Herelleviridae*, *Casjensviridae*, and *Drexlerviridae* had the highest representation among the 16 dominant families.

**Fig. 2:**
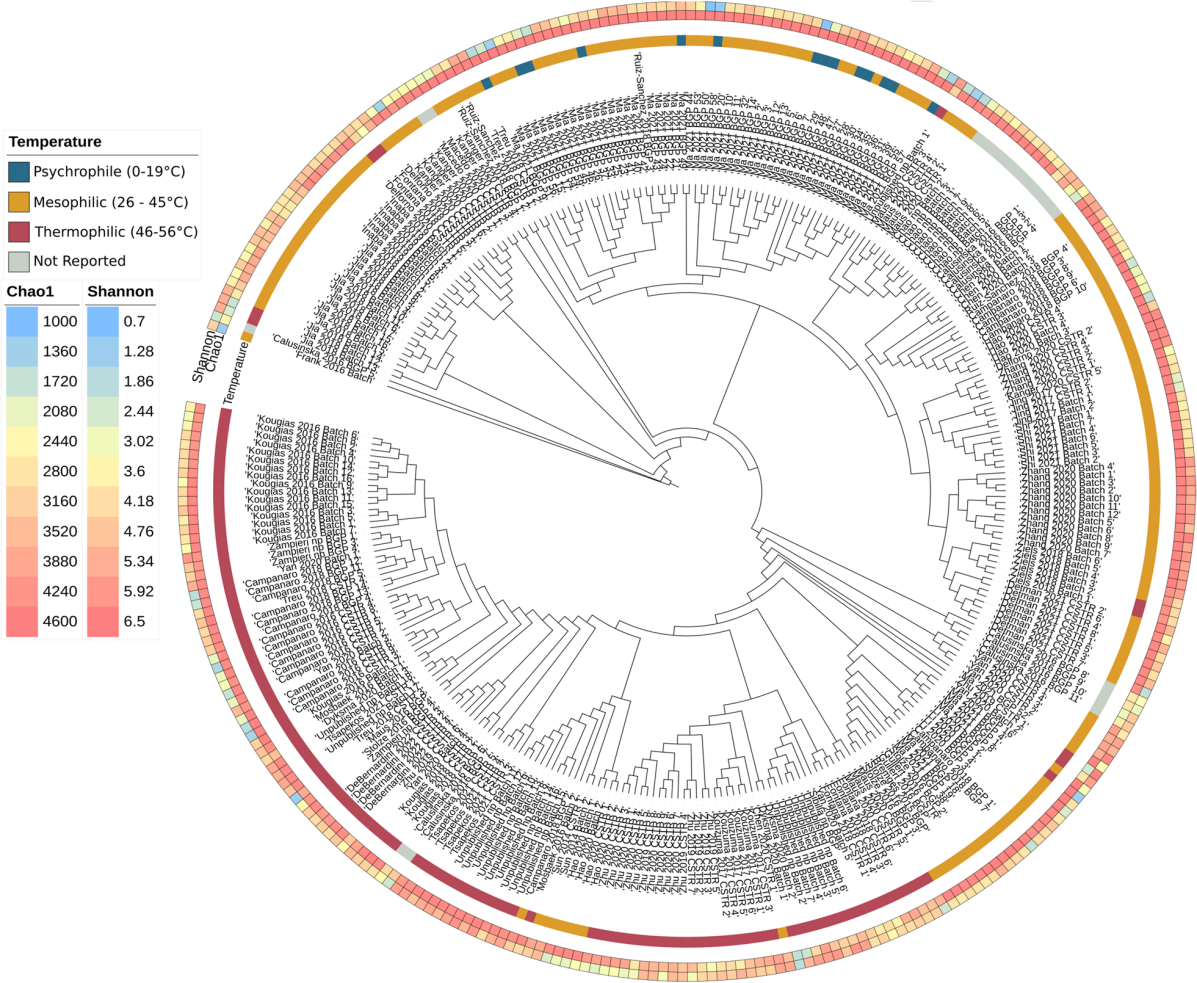
Microbial diversity of the expanded Biogas Microbiome database. Microbial community similarity across samples expressed by beta-diversity. Outer circles indicate the operational temperature and the community alpha-diversity as expressed by Chao1 and Shannon index

Detection of some evidence regarding previous infections can be used to study virus-host interactions. CRISPR spacers in particular were used to identify 2901 interactions between 546 MAGs and 1,822 viruses (Fig. 2A). The presence of shared spacers between phages and bacteria suggests a history of phage-bacterial interactions, and specifically lysogenic infections [66]. Most phages had only one host, but virMAG 384269_AS06 (*Herelleviridae* family) shared 40 different spacers with one bacterial MAG, *Candidatus* Anammoximicrobium sp. 06rmzA_251. Both have similar GC percentages (62.61–64.4) and are found exclusively in one experiment [67], suggesting they shared a long period of co-evolution, with the incorporation of new spacers in the bacteria and the ability to evade the recognition of CRISPR system by phages. In contrast, virMAG 27752_AS02 (family *Straboviridae*) was found in spacers of 8 different MAGs belonging to Firmicutes Phyla, order Clostridiales, with the exception of one MAG of Tissierellia class. This phage has a broader range of infection and can be present in different conditions, from samples with only blood agar (BA) medium and casein (Zhu_2019_CSTR_2) to complex feedstock such as raw municipal biowaste (Tsapekos_2021_CSTR_5). One of the most abundant phages (virMAG MPIBJC05F1_637440, family *Vertoviridae*) is present in 174 different samples from 9 countries and only two hosts were identified: Firmicutes sp. 28xzH2_85 and *Clostridiaceae* sp. 31mySI_51, with similar GC% and taxonomically close. Despite the specificity of this virus, the host’s widespread distribution allowed its spread in the AD system across different environmental conditions.

In the majority of the analyzed experiments, the archaeal abundance was lower than bacteria, which limited the detection of CRISPR. Despite this limitation, 76 archaeal genomes were identified to possess CRISPR arrays with more than 4 spacers. Further analysis of these genomes revealed that the genera *Methanothermobacter* and *Methanosarcina* were the most infected, accounting for 70% of all recorded virus-archaea interactions (Fig. 2B). Phages infecting *Methanothermobacter* and *Methanosarcina* archaea do not belong to the same family. In fact, those with the highest abundance infecting *Methanothermobacter* are found in a set of batch experiments (Kougias_2016_Batch) [68], while phages infecting *Methanosarcina* are less dominant but distributed across a greater number of samples, including batch experiments (Ma_2021_BGP_52) and biogas plants fed with sweet potato [21] and manure [12].

### Functional analysis of the microbial community

To understand the biological drivers of AD, functional annotation was integrated with taxonomic abundance, stratifying KEGG modules at different taxonomic levels. In order for a microbial consortium to perform a metabolic process, the simultaneous presence of all its functional units is required, though some gaps may exist in MAGs. As a result, only MAGs with complete and one-block-missing modules were considered (Additional file 3 for Archaea, Additional file 4 for Bacteria). Some functional modules are of particular interest in AD communities (Materials and methods, Taxonomic and functional prediction. Besides the obvious role of methanogenesis,beta-oxidation is relevant for fatty acid degradation when feedstocks are particularly rich in lipids. Additionally, modules involved in anaerobic carbon metabolismnitrogen metabolism, and sulfate reduction were also considered due to their influence on the AD process efficiency. and on the interactions between Bacteria and Archaea. For example, ammonia nitrification influences methane production since NH_4_ can strongly affect both the methanogenesis process, and the growth of methanogens, including for example *Methanobacterium*, *Methanosarcina,* and *Methanospirillum spp.* [69, 70].

### Archaeal community

**Fig. 3:**
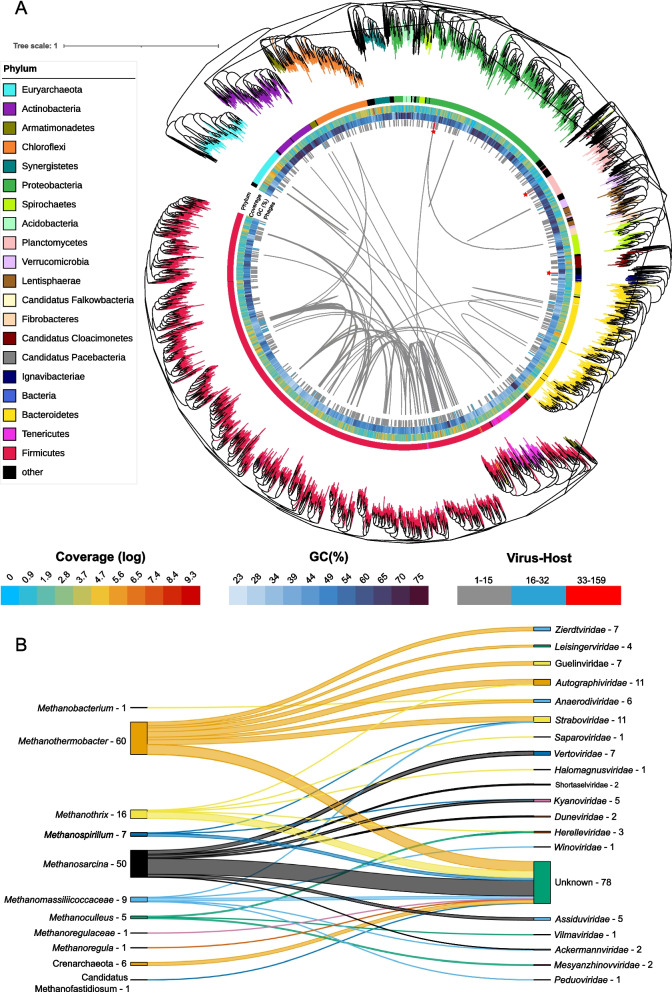
Phylogenetic tree and virome interaction. **A** The tree is represented in an inverted orientation, with branches color-coded according to phylum taxonomy. Legend is clockwise oriented starting from Euryarchaeota phylum The outermost ring exhibits the phylogenetic classification of the various taxa (Phylum), followed by subsequent rings that display the log-normalized coverage for each MAG (Coverage), the percentage of guanine-citosine (GC%), and the number of interactions with phages (Phages). Red asterisks indicate MAGs with more than 32 phage interaction signals. Phages that infect more than two MAGs are indicated by inner lines. **B** Relationships between different archaeal genera and the phages that infect them, the numbers indicate the total number of genomic sequences

**Fig. 4:**
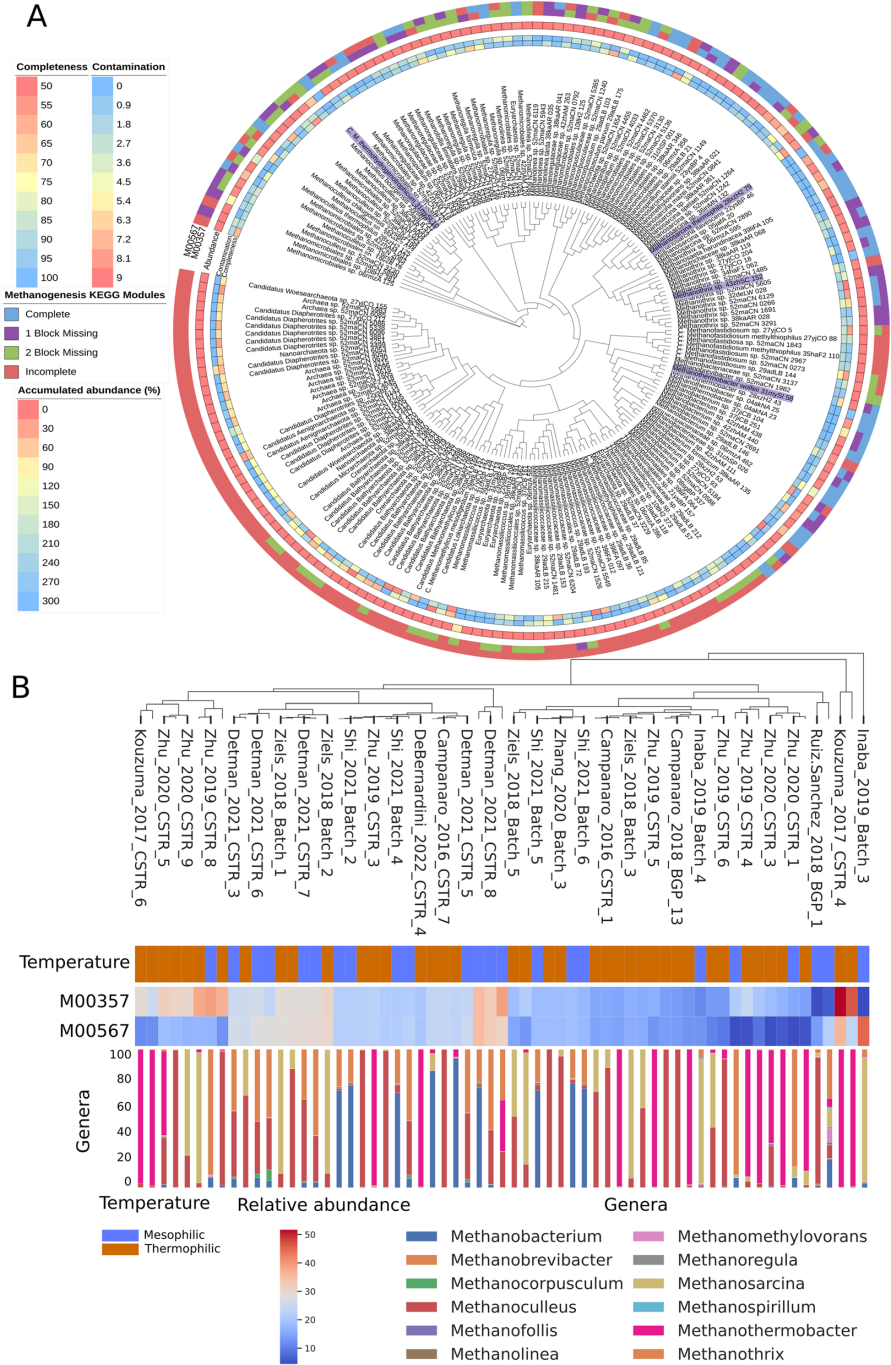
Methanogenesis modules in archaeal MAGs. **A** Phylophlan tree with all archaeal MAGs encoding the methanogenesis modules. MAGs quality (completeness and contamination) and their cumulative abundance (%) are displayed in the three innermost circles. The MAGs more frequently identified in the samples of the AD database are highlighted in light purple. **B** Heatmap reporting the samples with more than 10% relative abundance of complete and one block missing methanogenesis modules. The barplot represents the relative abundance of each genus in the samples and was calculated by taking into account only MAGs with complete modules, and those with one block missing. M00357: methanogenesis, acetate =  > methane; M00567: methanogenesis, CO_2_ =  > methane

Out of 198 archaeal MAGs, 70 (35%) have either complete or one missing block for methane production from acetate (M00357; acetoclastic methanogens), with most belonging to the class Methanomicrobia (72%), followed by Methanobacteria (24%). Considering methane production from CO_2_ (M00567; hydrogenotrophic methanogenesis), the proportion of Methanomicrobia is even higher, with 55 MAGs out of 72 (76%). Out of the total 89 MAGs with either one of the methane production modules, 72% have both methane production modules, of which 69% belong to the Methanomicrobia class and 19% to Methanobacteria. Both classes are reported in literature as hydrogenotrophic methanogens, which is the most widespread pathway in Archaea [71]. Except for *Candidatus* Methanoculleus thermohydrogenotrophicum 31mySI10, which has a well-defined taxonomic affiliation, all the *Candidatus* archaeal MAGs harbor incomplete (or two block missing) methanogenic modules (Fig. 3A), indicating the need for a more detailed functional investigation. Actually, a significant portion of genes found in archaea, ranging from approximately 30% to as high as 80%, code for proteins labeled as 'hypothetical proteins'. This is primarily due to the challenges in isolating and culturing most archaea in the laboratory, which makes experimental characterization of their gene repertoire difficult [72]. In contrast, the three most abundant archaeal MAGs in all the samples (*Candidatus* Methanoculleus thermohydrogenotrophicum 31mySI10, *Methanotrix sp.* 43zhSC152, and *Methanothermobacter wolfeii* 31mySI 58) have both modules complete. However, only *Methanothrix* exhibits both functional methane pathways in vivo experiments [73]. *Methanoculleus* and *Methanothermobacter* are known hydrogenotrophs [74], and there is no evidence that they use acetoclastic pathways. This emphasizes the necessity for specific archaea annotations to ensure more precise genomes characterization (Fig. 4).

The estimation of the relative abundance of each KEGG module in each sample was obtained by considering the complete modules and those with one block missing. According to this calculation, samples “Kouzuma_2017_CSTR_4” and “Shi_2021_Batch_1” had the highest values for methanogenesis KEGG modules (M00357, M00567) (Fig. 3B). In the “Shi_2021_Batch” experiment, hydrochar was used to enrich methanogenic species including *Methanobacterium*, *Methanolinea*, and *Methanothrix* genera, and increase methane production. Conversely, “Kouzuma_2017_CSTR_2 and 4” and most samples of “Zhu_2020_CSTR” had the highest abundance of acetoclastic functions, represented by *Methanosarcina*, and *Methanothermobacter* taxa. The presence of a diverse range of feedstock compositions can be observed in samples where methane production modules exhibit a relative abundance of over 10%, including acetate [4, 74–76], casein [75], glucose [75], oleate [67, 77], manure [67, 77–79], lactate [4], butyrate [4], propionate [4, 79], sludge [80], and municipal biowaste [81]. However, the most abundant samples with both modules [4, 74, 79] were fed with acetate and propionate.

The observed variations in methanogenesis and acetoclastic functions across different biogas reactors indicate a significant influence on the microbial community composition on biogas production. The enrichment of methanogenic species in certain samples, facilitated by specific experimental conditions such as the use of hydrochar, suggests a potential for increased methane production. The confirmation that reactors fed with organic acids, particularly acetate, enrich specific microbial species, irrespective of temperature conditions, suggests a strategic approach for optimizing biogas production. These findings emphasize the importance of tailoring substrate selection and reactor conditions to enhance the performance of microbial communities, providing valuable insights for improving overall biogas yields in anaerobic digestion processes.

### Bacterial community

The results obtained from the functional analysis indicate that three KEGG modules are widespread in the AD bacterial community: beta-oxidation (M00087), dissimilatory nitrate reduction to ammonium (M00530), and assimilatory sulfate reduction to sulfide (M00176) (Additional file 2: Fig. 4). The presence of the “dissimilatory nitrate reduction to ammonium” function in 71% of phyla suggests that denitrification is widespread in the AD community, and according to this, the two-step process of nitrate conversion to ammonia and finally to nitrogen through denitrification of nitrate to nitrogen module (M00529) is very common. Despite other studies reported that the bacterial dissimilatory nitrate reduction to ammonium pathway only dominates under low nitrate availability and in sulfide-free environments [82, 83], the present data suggest that species using nitrate (rather than oxygen) as electron acceptor are dominant in the AD microbiome. A previous study used a CSTR to remove linear alkylbenzene sulfonate (LAS) present in commercial laundry wastewater (Delforno_2020_CSTR_1); in this investigation a higher abundance of dissimilatory nitrate reduction to ammonium, denitrification, and beta-oxidation modules were identified (Additional file 2: Fig. 4). Beta-oxidation is the second step of LAS degradation and is mainly performed by *Synergistes* and *Syntrophus*, which are widespread genera in LAS degradation reactors [84–86]. Wastewater frequently contains several nitrogen-rich compounds, including nitrate, nitrite, and ammonia [87], which may have contributed to the enrichment of bacteria capable of converting nitrogen to different oxidation states.

Nitrogen metabolism was frequently identified in some taxa including the candidate Zixibacteria division and Acidobacteria. For example, half of the MAGs assigned to the candidate Zixibacteria division have the complete function of nitrification (M00528) and denitrification (M00529), reflecting their metabolic versatility [88]. Regarding the Acidobacteria phylum, nitrification (M00528) and complete nitrification (M00804) modules were identified in 50% and 25% of the MAGs, respectively; despite this finding the nitrification ability was not proven in isolates of the Acidobacteria phylum [89, 90]. The Acidobacteria nitrification function was more abundant in thermophilic manure-supplemented biogas plants with high biogas production and low pH (Campanaro_2018_BGP_3).

AD biogas production is heavily influenced by the organic substrates used. Some of these substrates may contain inhibitors such as sulfides, which can negatively affect the microbiome and decrease the AD process efficiency [91]. For example, sulfate-reducing bacteria (SRB) encoding proteins involved in the assimilatory sulfate reduction to sulfide function can compete with hydrogenotrophic archaea for hydrogen, and generate H_2_S as final product [65, 66]. In particular, a batch reactor fed with cellulosic and xylan biomass (Jia_2018_Batch) showed the highest representation of assimilatory sulfate reduction to sulfide across MAGs. Although the H_2_S concentration was not reported, all the batch experiments produced low concentration of CH_4_ (0.2–1.5 mM), suggesting that the process predominantly shifted to sulfate reduction [55]. Indeed, 68% of “Jia_2018_Batch” library read counts mapped to the *Clostridium butyricum* 37jiCB_291 MAG, a known SRB [83]. Overall, a global Biogas Microbiome database can be useful to infer putative inhibitory in the AD process by analyzing the pathways of the MAGs identified.

In contrast, key carbon metabolism modules including the Arnon-Buchanan cycle, the WL pathway, and the acetogenesis are complete or have only one block missing in less than 25% of the total MAGs suggesting these are shell modules in the AD system [92]. Nevertheless, these metabolic routes for carbon were identified in a range of phyla including Actinobacteria, Chloroflexi, Firmicutes, Ignavibacteriae, Proteobacteria, and Spirochaetes. The Arnon-Buchanan cycle module, involving a reverse citric acid cycle for CO_2_ fixation, was the most abundant in samples inoculated with sludge (Macedo_2020_CSTR and Zhang_2020_Batch), while the WL and acetogen modules were more abundant in a CSTR sample for the AD of saccharides with a feedstock of volatile fatty acid mixture (Zhu_2019_CSTR_5).

### Microbial replication rates are linked to their functional capabilities

**Fig. 5:**
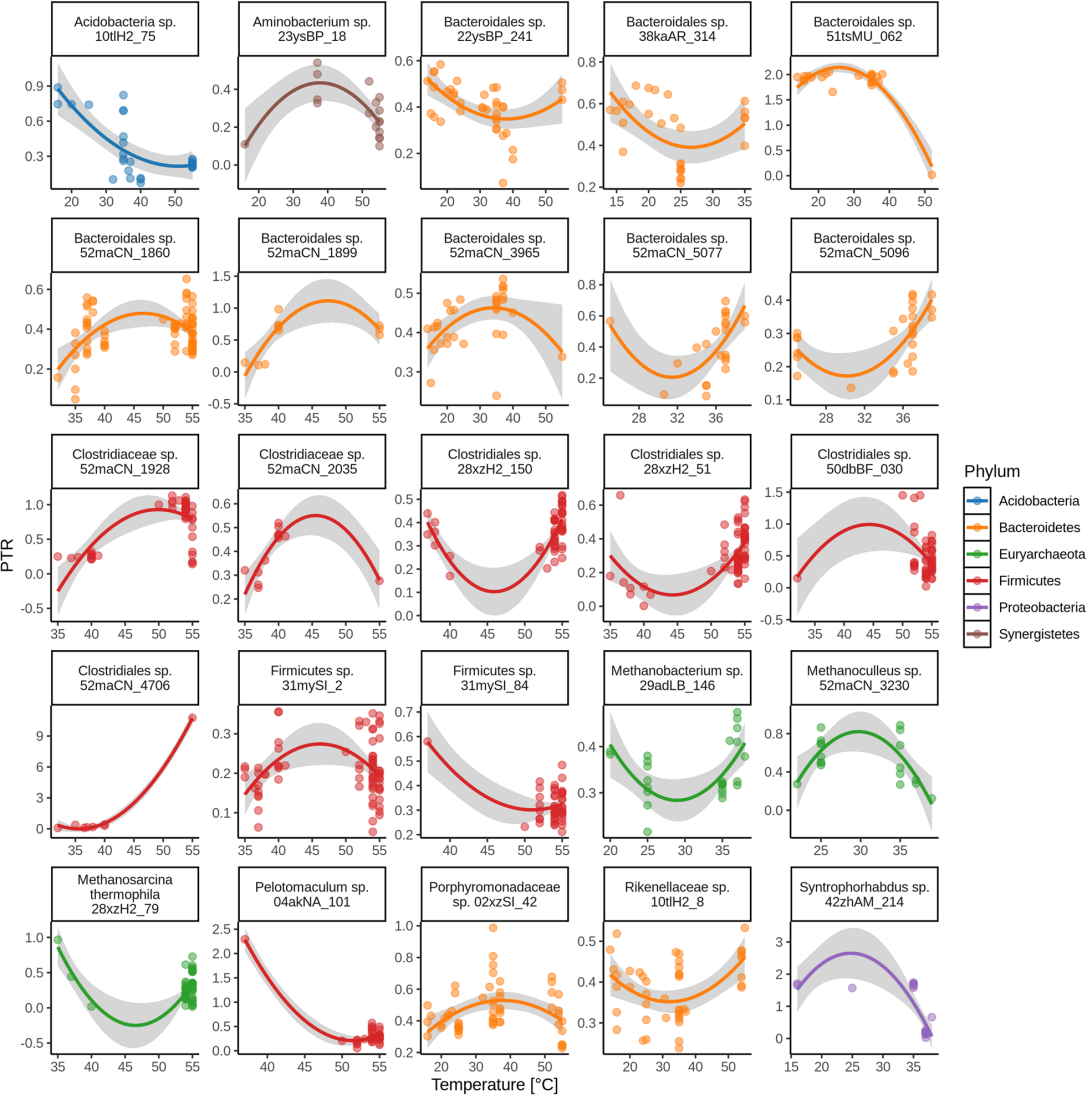
Relationship between PTR and temperature for individual MAGs. Shown values refer to significant relationships as assessed by quadratic polynomial fits, based on a FDR-adjusted *p*-value threshold of 0.1

To characterize microbial dynamics across AD systems, we calculated the peak-to-trough ratio (PTR) of the MAGs coverage, which estimates DNA synthesis and generation rate [47]. Sample-specific PTRs were obtained for 782 MAGs in the dataset, corresponding to those genomes meeting the minimal coverage requirement in at least one sample. As a result, PTR values were determined in fewer than 20 samples for most MAGs, while for others, e.g. *Methanothrix* sp. 43zhSC_152, Synergistaceae sp. 24abBP_148, Bacteroidales sp. 28xzH2_30, and various members of Firmicutes, PTRs were estimated for over a hundred samples, reflecting their widespread abundance. Resulting PTR values generally have a long-tail distribution, with a median of 0.37 and exceeding 2 in some cases, with varying spread across different taxonomic groups (Additional file 2: Fig. 5). Within these distributions, both coarse- and fine-grain trends matching previous knowledge were observed. Firstly, acetoclastic and hydrogenotrophic methanogens exhibited a low mean PTR, consistently with direct measures of the replication rate in isolates (Additional file 2: Fig. 6). Secondly, slightly lower mean PTR was observed in batch reactors, consistently with the limited duration and efficiency of the processes therein compared to CSTRs and full-scale plants. When considering individual microorganisms, significant PTR trends for MAGs classified at genus or species level are in some cases in agreement with documented growth temperature preferences, where robust PTR distributions could be recovered (Fig. 5) [93, 94]. In particular, microorganisms of the *Methanoculleus* genus are cultivated at a temperature between 28° and 37 °C, and *Methanoculleus* sp. 52maCN_3230 has an estimated optimal growth temperature in approximately the same interval. *Porphyromonadaceae* sp. 02xzSI_42 reaches the highest PTR values around 40 °C, which is its recommended cultivation temperature [93]. Similarly, *Methanosarcina thermophila* 28xzH2_79 has detectable non-null PTR values predominantly close to 55 °C, even though some outliers were found in the mesophilic range. PTR distribution for other species, including *Methanobacterium* sp. 29adLB_146, *Aminobacterium* sp. 23ysBP_18, and *Syntrophorhabdus* sp. 42zhAM_214, show their maximum values in the 25–40 °C range, in agreement with the largely mesophilic representation of these genera [94]. Although replication rate is generally influenced by a variety of factors, together, these patterns support the overall soundness of obtained distributions.

Besides, other less obvious trends were found. While Verrucomicrobia, Fibrobacteres, and Planctomycetes are among the phyla with the largest mean PTR, subsets of Proteobacteria and Bacteroidetes show a more noticeable long tail around large values (Additional file 2: Fig. 5). More specifically, MAGs classified as Syntrophobacterales seem to have fast replication rates in several conditions. This taxon harbors sulfate reducers, which can efficiently metabolize substrates such as pyruvate, methanol, and glucose and outcompete methanogens when sufficient sulfate is available [95, 96]. These results are confirmed by the long tail of PTRs associated with assimilatory sulfate reduction (Additional file 2: Fig. 6), and thus support the rapid proliferation capability of these microbes. Other of such fast-growing microbial groups are Bacteroidales (e.g. *Proteiniphilum* sp. 29adLB_192), with relevant proteolytic role [97], and microbes involved in fatty acid oxidation (Additional file 2: Fig. 7). These groups could represent species with high energy generation capability from the breakdown of such macromolecules. Moreover, archaeal phyla also have a wide range of replication rates, with some MAGs exhibiting high PTRs. In particular, Methanomicrobiales sp. 21ysBP_11 and *Methanosarcina flavescens* 22ysBP_46 are among the fast-growing archaea with PTR consistently above 2, making Methanomicrobiales sp. 21ysBP_11 a potential promising candidate for cultivation and isolation. In general, most MAGs exhibit their highest replication rates within a 5 °C range (preferential temperature range), indicating that temperature tightly controls replication efficiency (Additional file 2: Fig. 7). In fact, mesophilic and thermophilic communities tend to present distinct composition and diversity, as also seen above. Yet, a relevant number of bacterial and archaeal MAGs show their highest PTRs over a 20 °C window, suggesting that some species are able to better adapt across temperature regimes.

### Numerous genomic variants delineate microbial population heterogeneity

To date, there is a lack of information in literature regarding the genetic heterogeneity of AD-relevant species [74]. The presence of the same microbial species in reactors characterized by different process parameters can allow to identify variants impacting the adaptation process, as well as a more detailed characterization of species at strain level. Variant identification was performed by aligning shotgun reads of each experiment back to the MAGs obtained from the binning process, and this approach led to the identification of 10.5 millions single nucleotide variants (SNVs). The high number of variants revealed a high genetic heterogeneity in the microbial population, and variants characterization allowed their classification as synonymous (60.5%), nonsynonymous (28.9%), intergenic (10.4%), and multigenic (0.02%). Of the 3,050 MAGs containing SNVs, only eight exhibited more than 500,000 SNVs, two of which were the methanogenic archaea *Methanothrix* sp. 43zhSC_152 and *Methanoculleus* sp. 52maCN_3230, which harbor 938,000 and 661,000 SNVs, respectively (see Availability of Data and Materials). The high number of variants observed in *Methanothrix* sp. 43zhSC_152 could be due to the fact that this MAG is widespread, being present in 96 of the examined samples; possibly, the presence of this species in many reactors with highly different conditions have led to the differentiation of a large number of strains harboring genetic variants. On the other hand, the presence of *Methanoculleus* sp. 52maCN_3230 was limited to 23 mesophilic samples, and thus, the high number of genetic variants observed in this MAG could be due to different factors in comparison to *Methanotrix*.

**Fig. 6:**
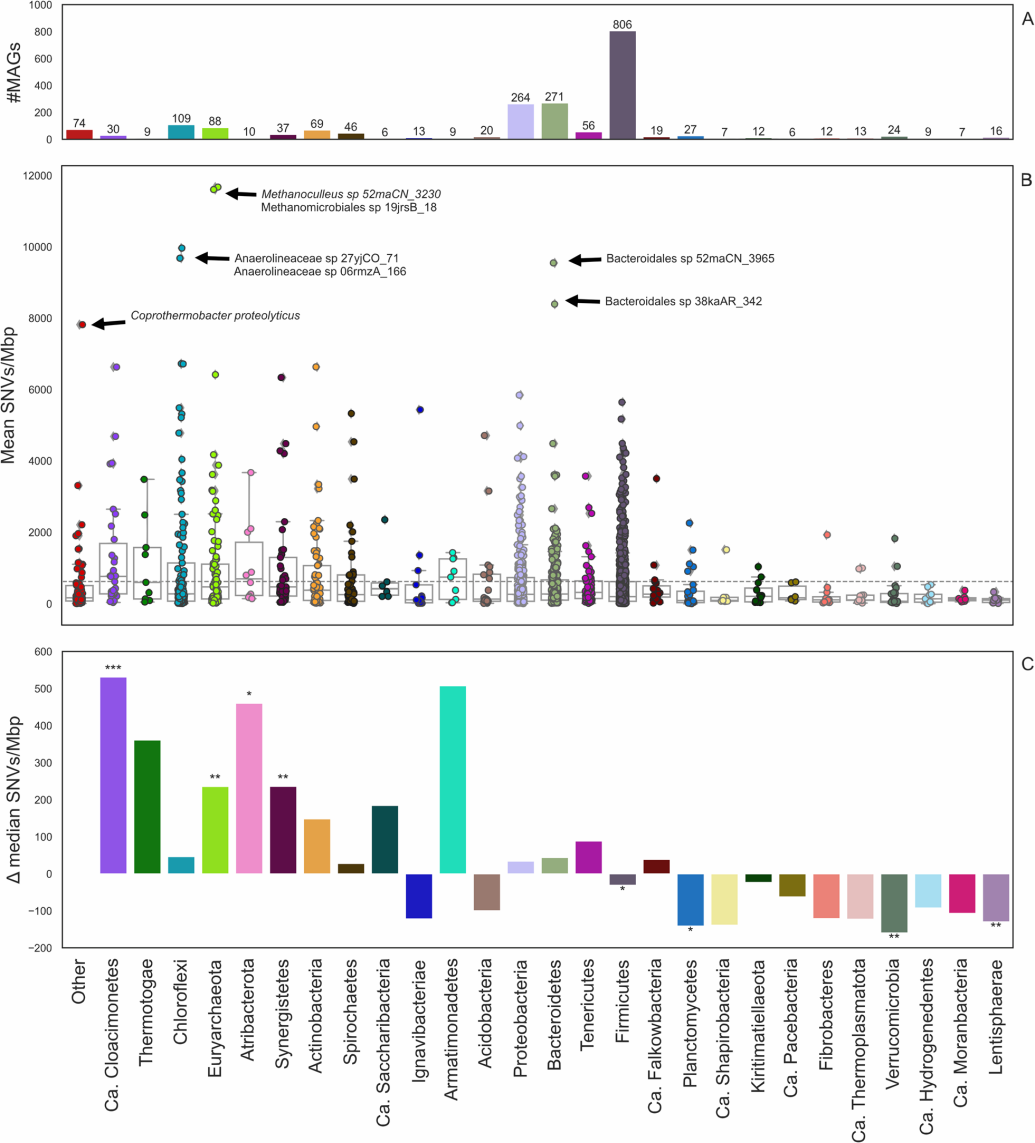
Overview of variants distribution among different taxonomic groups. **A** Number of MAGs associated with each phylum. **B** Number of SNVs/Mbp in each phylum for MAGs with more than 100 SNVs; each dot represents a MAG. Results are reported for each phylum with more than five MAGs, while all the others are reported as “Other”. **C** Statistical analysis comparing the median SNV density calculated for each phylum and the average value reported for the global Biogas Microbiome database. Statistically significant results are marked with asterisks: “*”*p* <  = 0.05, “**”*p* <  = 0.01, “***”*p* <  = 0.001 and “****”*p* <  = 0.0001

In order to obtain a more reliable representation of the genomic variability, the number of SNV per MAG was normalized, both according to the genome length and to the number of samples where the MAG was identified with coverage higher than 1 (Fig. 6B). This analysis highlighted *Methanoculleus* sp. 52maCN_3230 and *Methanomicrobiales* sp. 19jrsB_18 as outliers with more than 10,000 SNVs/Mbp per sample (Fig. 6B). This finding is of particular interest because, while *Methanoculleus* sp. 52maCN_3230 is quite common in the AD samples, *Methanomicrobiales* sp. 19jrsB_18 was identified in two samples only. The genomic location of nonsynonymous variants identified in both methanogens allowed linking them to the gene and to the functional pathways. Interestingly, 2.5% of the *Methanomicrobiales* sp. 19jrsB_18 variants and 9.1% of the *Methanoculleus* sp. 52maCN_3230 variants were associated with core genes of the hydrogenotrophic methanogenesis, including heterodisulfide reductase (*hdr*), methyl-coenzyme M reductase (*mcr*) and formylmethanofuran dehydrogenase (*fwd*). Overall, these results suggest that variants with a crucial role in the adaptation of methanogenic archaea to reactors operated in different conditions.

In order to determine if some phyla were statistically more impacted by variants, the taxonomic assignment of each MAG at phylum level was considered along with the number of SNVs/Mbp. Results of the Mann–Whitney U-test on SNVs/Mbp distribution showed that eight phyla had a significant enrichment in the number of variants (*p*-value < 0.05) (Fig. 6C), with *Candidatus* Cloacimonetes (*p* = 0.0003), Euryarchaeota (*p* = 0.0025), Atribacterota (*p* = 0.0307) and Synergistetes (*p* = 0,0023), having a number higher than expected, and Firmicutes (*p* = 0.0380), Planctomycetes (*p* = 0.0145), Verrucomicrobia (*p* = 0.0043) and Lentisphaerae (*p* = 0.0015), having a lower number. In general, the results obtained for Euryarchaeota suggest that methanogenic archaea are under a strong selective pressure and harbor a large amount of genetic variability. The metabolic roles of species belonging to *Candidatus Cloacimonetes* and *Synergistetes* are still not completely clear, however, it was previously reported that some of them are characterized by acetogenesis [98], or can compete for acetate utilization with *Methanosaeta* [99].

To get a first glimpse on the strains composition, a phylogenetic analysis was conducted on *Methanothrix* sp. 43zhSC_152, *Methanothermobacter wolfeii* 31mySI 58 and *Candidatus Methanocullus* thermohydrogenotrophicum 31mySI_10, the three archeal species with the highest number of MAGs. These three species have different properties in the database: *Methanotrix* has a high number of MAGs and a worldwide distribution, the other two have less MAGs and are more abundant in European reactors (see Availability of Data and Materials). Phylogenetic analysis revealed a regional distribution of MAGs for *Methanotrix* with a distinct cluster of MAGs deriving from Chinese biogas plants. Interestingly, all the MAGs recovered for *Methanothrix* sp. 43zhSC_152 are mesophilic, and all the MAGs of *M. wolfeii* 31mySI 58 are thermophilic, while *Candidatus* M. thermohydrogenotrophicum 31mySI_10 is more flexible and is present in some mesophilic and thermophilic samples (Additional file 2: Fig. 8). This finding contradicts previous data reporting this species only in thermophilic conditions [100], and suggests a different habit for this methanogen, which is able to adapt to mesophilic conditions as well. The temperature is not the main driver of strains differentiation for *Candidatus* M. thermohydrogenotrophicum, while the high phylogenetic distance among some *M. wolfeii* and *Ca.* M. thermohydrogenotrophicum MAGs suggest a possible impact of environmental conditions on their strains differentiation. Two *M. wolfeii* MAGs identified in samples subjected to high H_2_ concentrations, 15tlH2_55 and 50dbBF_040, appeared quite distant from the others suggesting a selective pressure determined by environmental conditions.

## Conclusions

In this study, the metagenomic characterization of the microbial species involved in the AD process has been expanded through the analysis of a large number of different reactor types operated under a range of conditions. In addition to expanding the number of species reported in the previous version of the Biogas Microbiome database by almost three times, the analysis was focused on archaea, one of the crucial components of the microbiome. The investigation of gene composition has led to a better characterization of archaea and their methanogenic metabolism; however there still remains a degree of uncertainty in the automatic association between gene composition and phenotype, which will require the development of new investigation methods based on gene expression or machine learning. Despite this huge increase in the number of catalogued species, the great diversity of this biotechnological niche has yet to be fully explored, especially with regard to bacterial species.

Inspection of the phage repertoire provided a first overview and, through the analysis of the CRISPR elements, a first characterization of phage-bacterial interactions and co-evolution. This analysis allowed to build the first version of the viral Biogas Microbiome database, currently represented only by DNA phages. The RNA phage fraction still remains to be identified and will be one of the next targets. While the viral component characterisation proved to be extremely complex, the presence of a combined database of prokaryotes and phages will certainly allow in the future a better tracking of their interactions with prokaryotes, also via means of cross-linking techniques and co-occurrence.

Abundance of microbial species competing with methanogens for H_2_ utilization, such as SRBs, has highlighted how a well-characterized MAG database allows to better understand the impact of the microbiome in reducing the performance in terms of biogas production. Finally, the investigation of SNVs impacting the genes involved in key functional processes has laid the foundations to study the evolution of AD microbiome and its role in reactor performance, suggesting that methanogenic archaea are under strong selective pressure.

### Supplementary Information

**Additional file 1**. Datasets and metadata information, assembly statistics and diversity values

**Additional file 2**. **Figure S1. **Relationship between alpha-diversity and temperature. **Figure S2. **Phylum distribution across samples.** Figure S3.** Phage abundance overview.** Figure S4. **Bacterial functional modules. **Figure S5.** PTR distribution across phyla.** Figure S6. **PTR distribution in main functional modules. **Figure S7.** Strain distribution in the main methanogens. **Figure S8. **Phylogenetic analysis of the MAGs identified for three of the most common methanogens. **Supplementary Figure S9.** GC content distribution across phage families.

**Additional file 3**. Archaeal MAGs with complete and one-block-missing M00357 and M00567 and their relative abundance across samples.

**Additional file 4**. Bacterial MAGs with complete and one-block-missing modules for selected functions and their relative abundance. For every considered function, the cumulative abundance of all the MAGs satisfying these criteria within any identified phylum is reported for each sample.

## Data Availability

The generated data represent the third stage of a continued effort to fully characterize the anaerobic digestion microbiome and make it fully available online. To generate version V1 and V2 of the MAGs database, shotgun sequences were previously described in prior studies [15, 77]. In the present study, shotgun sequences used were downloaded from SRA, and information associated with the projects is reported in Additional file 1Additional file 1. The MAGs and virMAG sequences are available through the “Biogas Microbiome V3” project on figshare with https://doi.org/10.6084/m9.figshare.22606030 (bacteria and archaea—https://doi.org/10.6084/m9.figshare.22606030) and 10.6084/m9.figshare.22626886 (viruses—https://doi.org/10.6084/m9.figshare.22626886) (https://figshare.com/). Full quality, taxonomy, and associated data of each MAG and virMAG are available at the corresponding web pages.

## Abbreviations

AD: Anaerobic digestion
MAGs: Metagenome-assembled genomes
KEGG: Kyoto Encyclopedia of Genes and Genomes
PTR: Peak-to-trough ratio
CSTR: Continuous stirred-tank reactor
CRISPR: Clustered regularly interspaced short palindromic repeats
PFAM: Protein families database
COG: Clusters of Orthologous Groups of proteins database
BA: Blood Agar
WL: Wood-Ljungdahl pathway
LAS: Remove linear alkylbenzene sulfonate
SRB: Sulfate-reducing bacteria
SNV: Single nucleotide variants
