# Supplementary material for: A unified compendium of prokaryotic and viral genomes from over 300 anaerobic digestion microbiomes
Source: Environ Microbiome. 2024 Jan 2;19:1. doi: 10.1186/s40793-023-00545-2 (PMC10762816; doi:10.1186/s40793-023-00545-2)
Supplement: Supplementary file 2 — Additional file 2. Figure S1. Relationship between alpha-diversity and temperature. Figure S2. Phylum distribution across samples. Figure S3. Phage abundance overview. Figure S4. Bacterial functional modules. Figure S5. PTR distribution across phyla. Figure S6. PTR distribution in main functional modules. Figure S7. Strain distribution in the main methanogens. Figure S8. Phylogenetic analysis of the MAGs identified for three of the most common methanogens. Supplementary Figure S9. GC content distribution across phage families. [file 40793_2023_545_MOESM2_ESM.docx]

**A unified compendium of prokaryotic and viral genomes from over 300 anaerobic digestion microbiomes**

Victor Borin Centurion^1^, Alessandro Rossi^1^, Esteban Orellana^1^, Gabriele Ghiotto^1^, Balázs Kakuk^2^, Maria Silvia Morlino^1^, Arianna Basile^3^, Guido Zampieri^1*^, Laura Treu^1*^, Stefano Campanaro^1^

**^1^** Department of Biology, University of Padua, via U. Bassi 58/b, 35131 Padova, Italy.

**^2^** Department of Medical Biology, Albert Szent-Györgyi Medical School, University of Szeged, 12 Somogyi B. u. 4., 6720 Szeged, Hungary

^3^ MRC Toxicology Unit, University of Cambridge, Gleeson Building Tennis Court Road Cambridge, UK

*Correspondence Author: [laura.treu@unipd.it](mailto:laura.treu@unipd.it), [guido.zampieri@unipd.it](mailto:guido.zampieri@unipd.it)

**List of figures:**

**Supplementary Figure 1.** Relationship between alpha-diversity and temperature.

**Supplementary Figure 2.** Phylum distribution across samples.

**Supplementary Figure 3.** Phage abundance overview.

**Supplementary Figure 4**. Bacterial functional modules.

**Supplementary Figure 5.** PTR distribution across phyla.

**Supplementary Figure 6.** PTR distribution in the main functional modules.

**Supplementary Figure 7.** Strain distribution in the main methanogens.

**Supplementary Figure 8.** Phylogenetic analysis of the MAGs identified for three of the most common methanogens.

**Supplementary Figure 9.** GC content distribution across phage families.


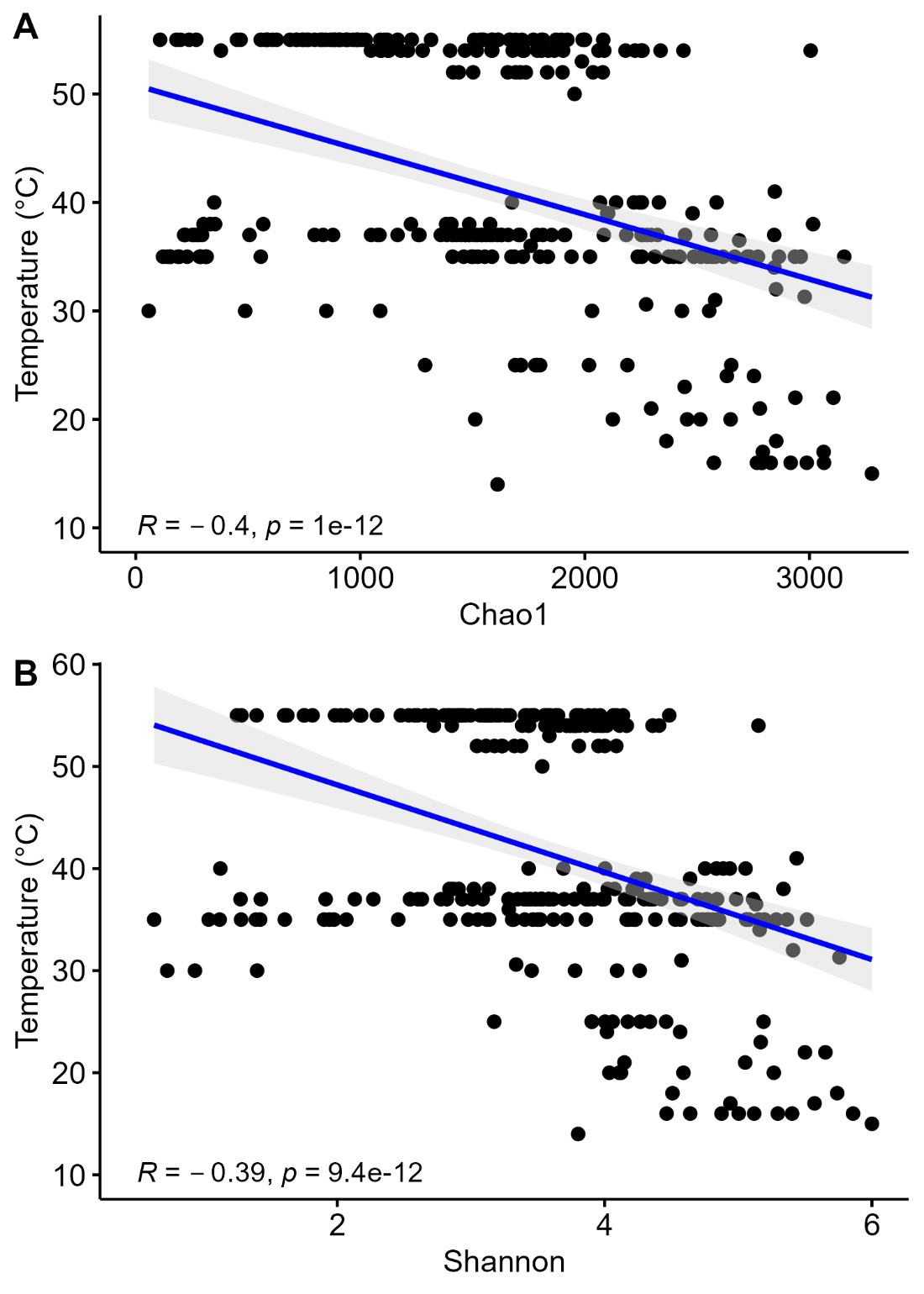


**Supplementary Figure 1. Relationship between alpha-diversity and temperature.** Pearson correlation of diversity indices Chao1 versus the temperature (ºC) **(A)** and Shannon indices versus temperature (ºC) **(B)**.


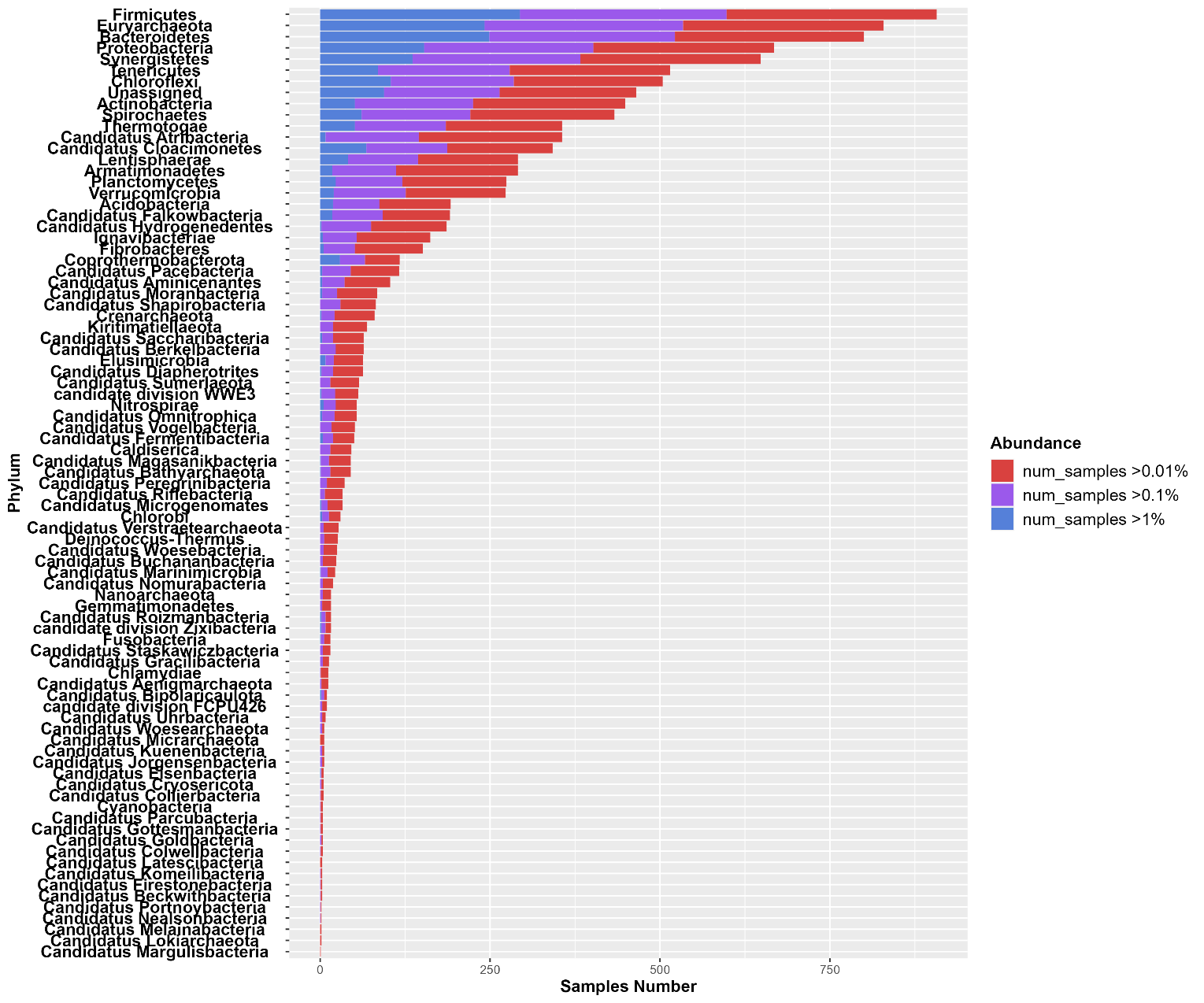


**Supplementary Figure 2. Phylum distribution across samples.** The varying colors indicate the relative abundance within each sample, with the fill representing the cumulative occurrence of that particular abundance across multiple samples.


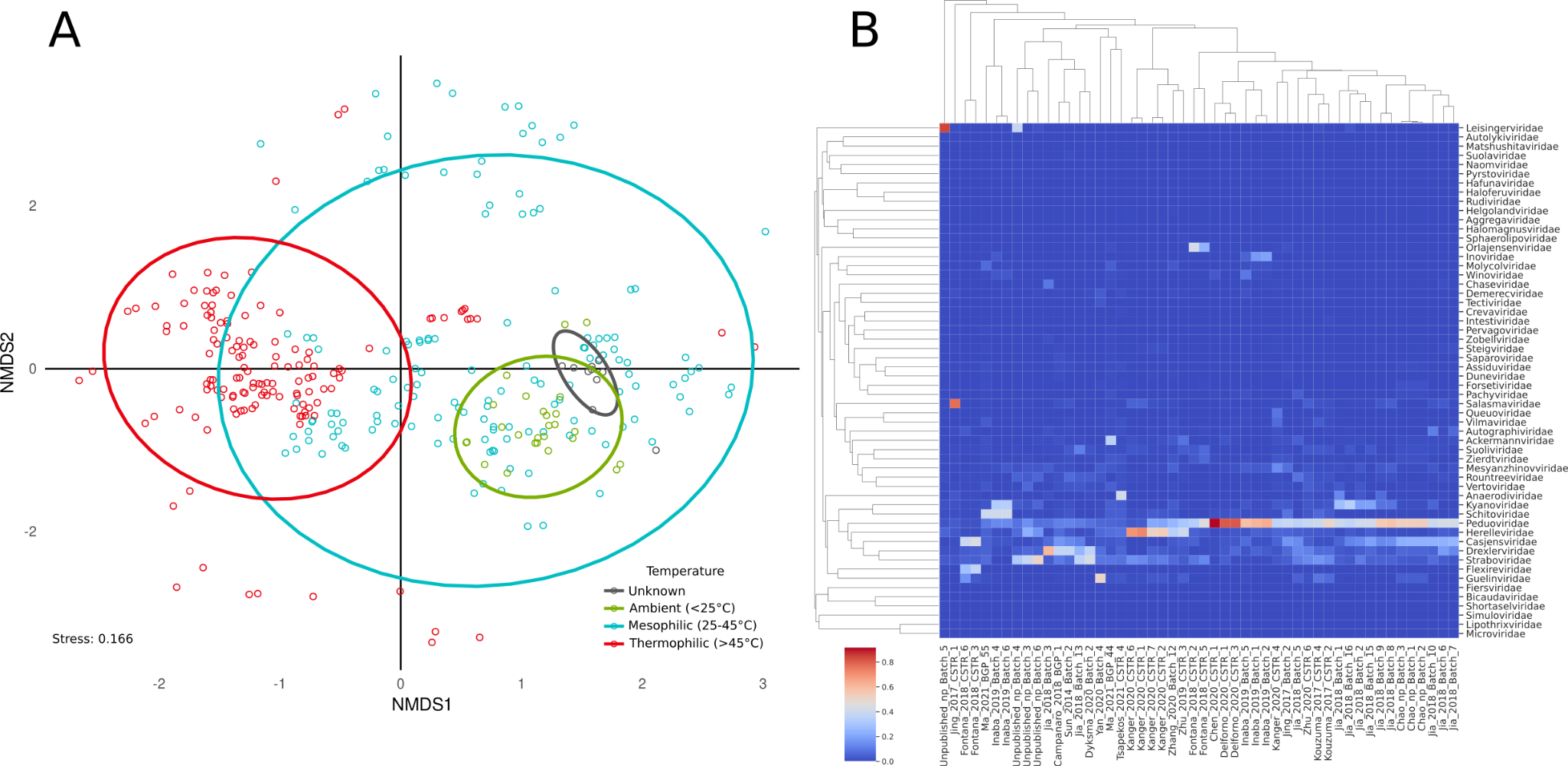


**Supplementary Figure 3. Phage abundance overview.** (A) Ordination diagram of the viral community in the experiment based on Bray-Curtis dissimilarities by non-metric multidimensional scaling (NMDS). Ellipses were drawn assuming a multivariate t-distribution based on reactor temperature. (B) Clustermap based on the abundance of each phage family in the 50 most abundant samples. The study Calusinska_2016_BGP [[12]](https://www.zotero.org/google-docs/?30HAaO) was discarded since it was focussed on extraction of phages.


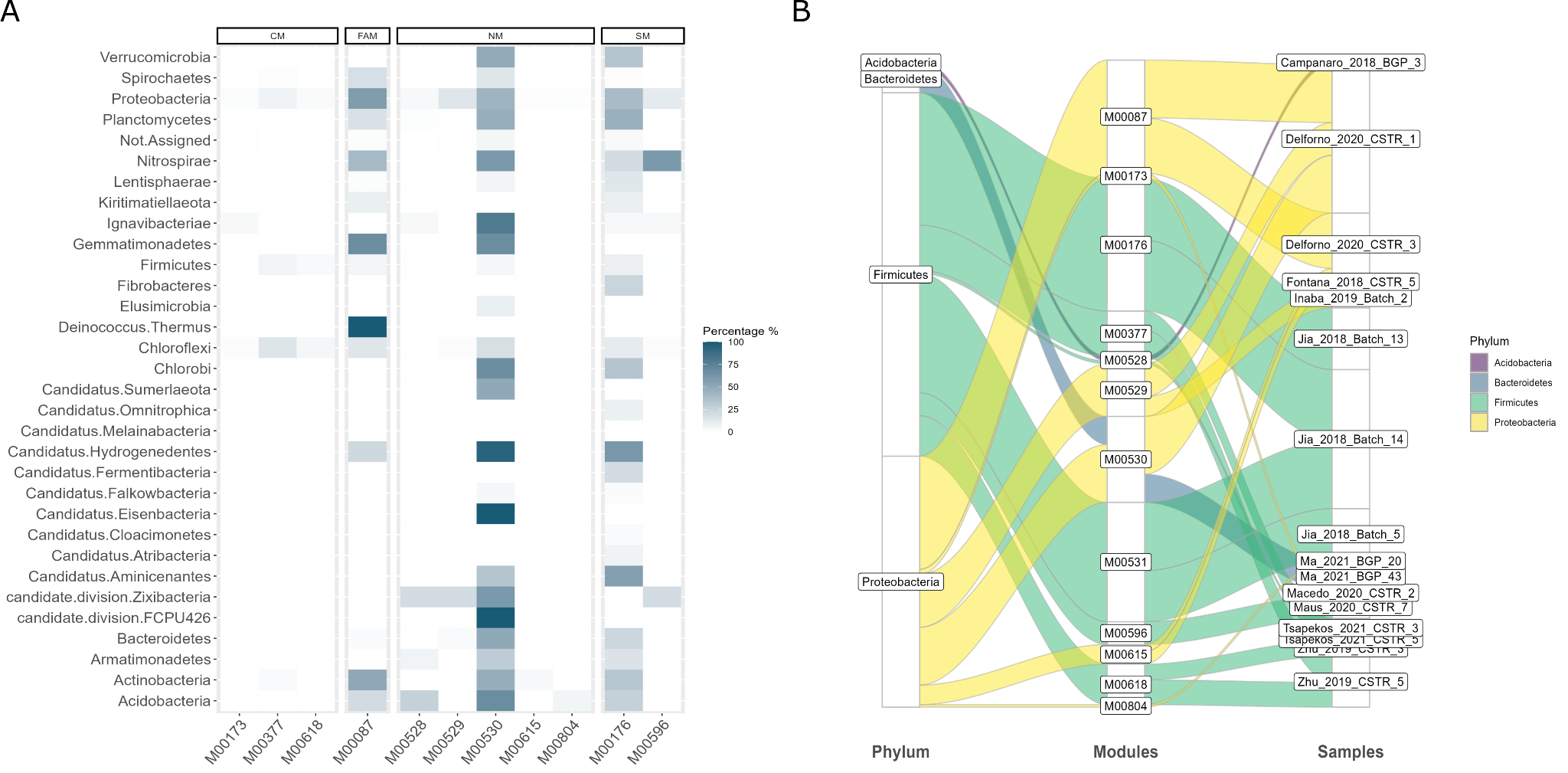


**Supplementary Figure 4. Bacterial Functional Modules.** Heatmap Plot (A): MAGs percentage classified at phylum level with complete or one block missing KEGG modules associated with the following functions: (1) carbon metabolism (CM; M00173, Arnon-Buchanan cycle; M00377, Wood-Ljungdahl pathway; M00618, Acetogen), (2) fatty acid metabolism (FAM; M00087, Beta-Oxidation), (3) nitrogen metabolism (NM; M00528, Nitrification, ammonia => nitrite; M00529, Denitrification, nitrate => nitrogen; M00530, Dissimilatory nitrate reduction, nitrate => ammonia; M00615, Nitrate assimilation; M00804, Complete nitrification, comammox, ammonia => nitrite => nitrate), and (4) sulfate reduction (M00176, Assimilatory sulfate reduction, sulfate => H2S; M00596, Dissimilatory sulfate reduction, sulfate => H2S). Alluvial Plot (B) showing for each KEGG module (central part) reported in the heatmap plot the two most representative samples (right), and their respective taxonomic information at phylum level (left).


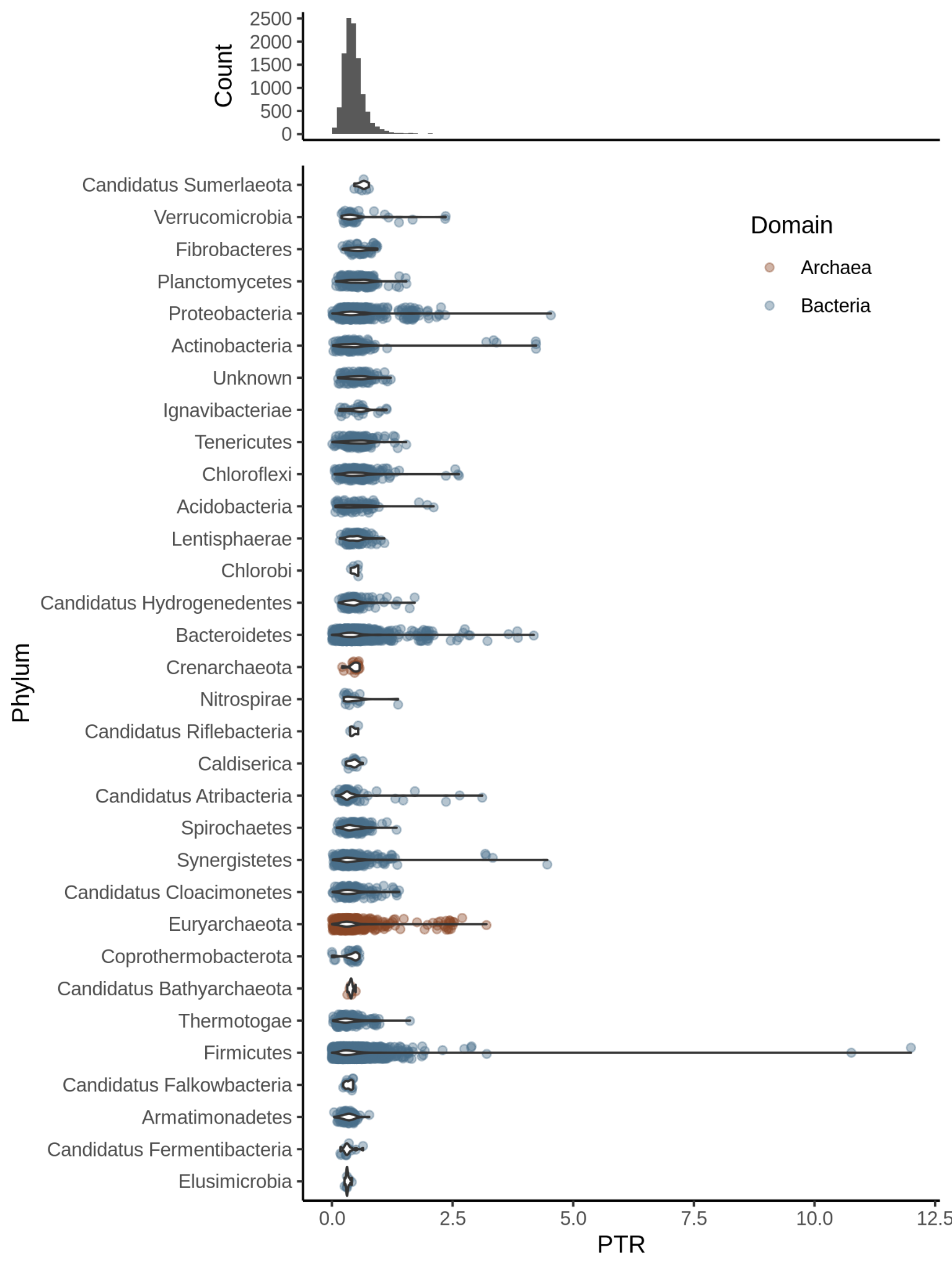


**Supplementary Figure 5. PTR distribution across phyla.** For each phylum, PTR values of the corresponding MAGs across the full sample set, ordered by decreasing average PTR.


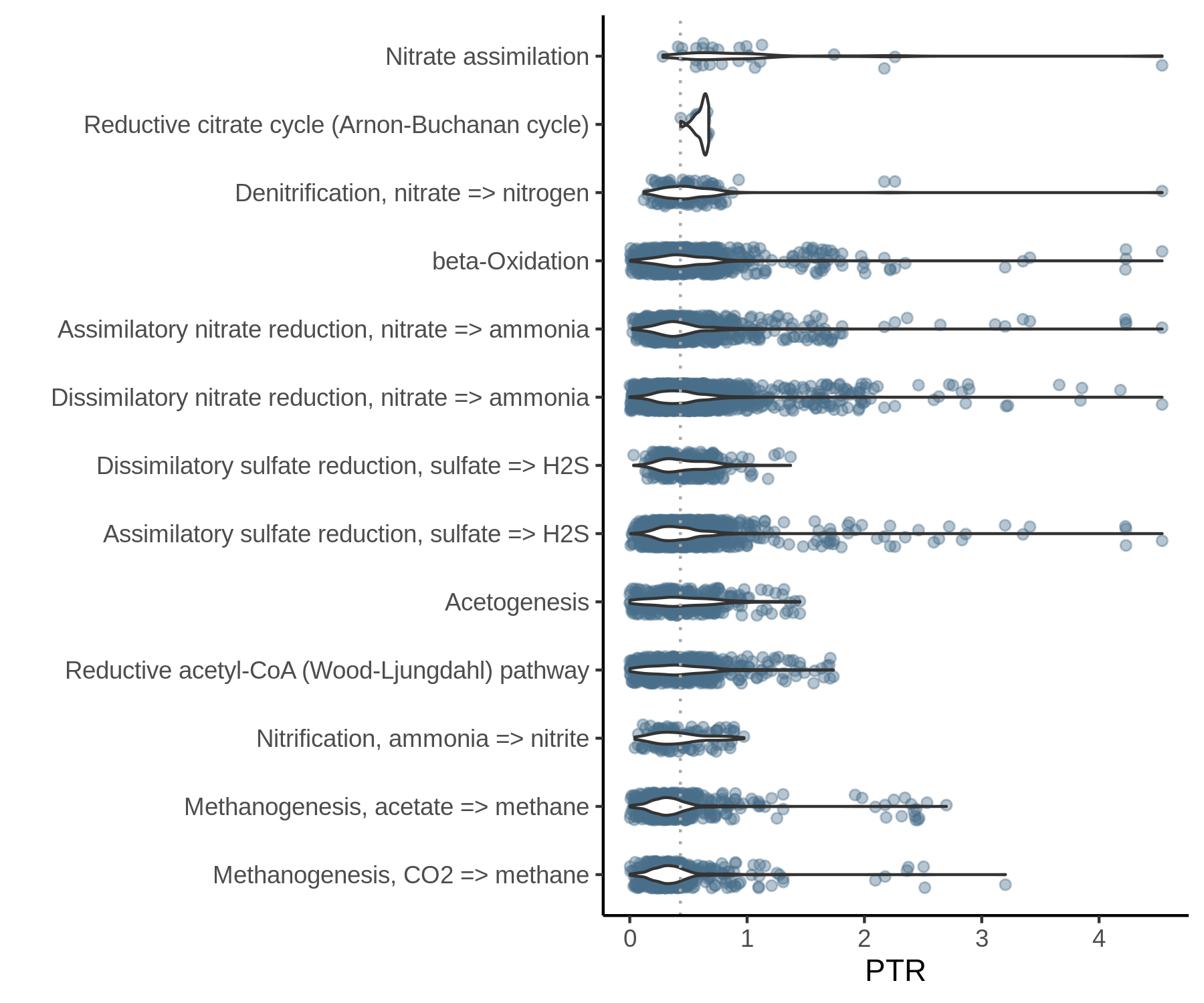


**Supplementary Figure 6. PTR distribution in the main functional modules.** For each module, the PTRs of MAGs having the module complete or missing a single KO are shown, ordered by decreasing average PTR. The dotted line represents the global PTR mean. MAGs associated with nitrate assimilation and reductive citrate cycle make up the groups with the average largest PTR, while methanogenic archaea have the average lowest values.


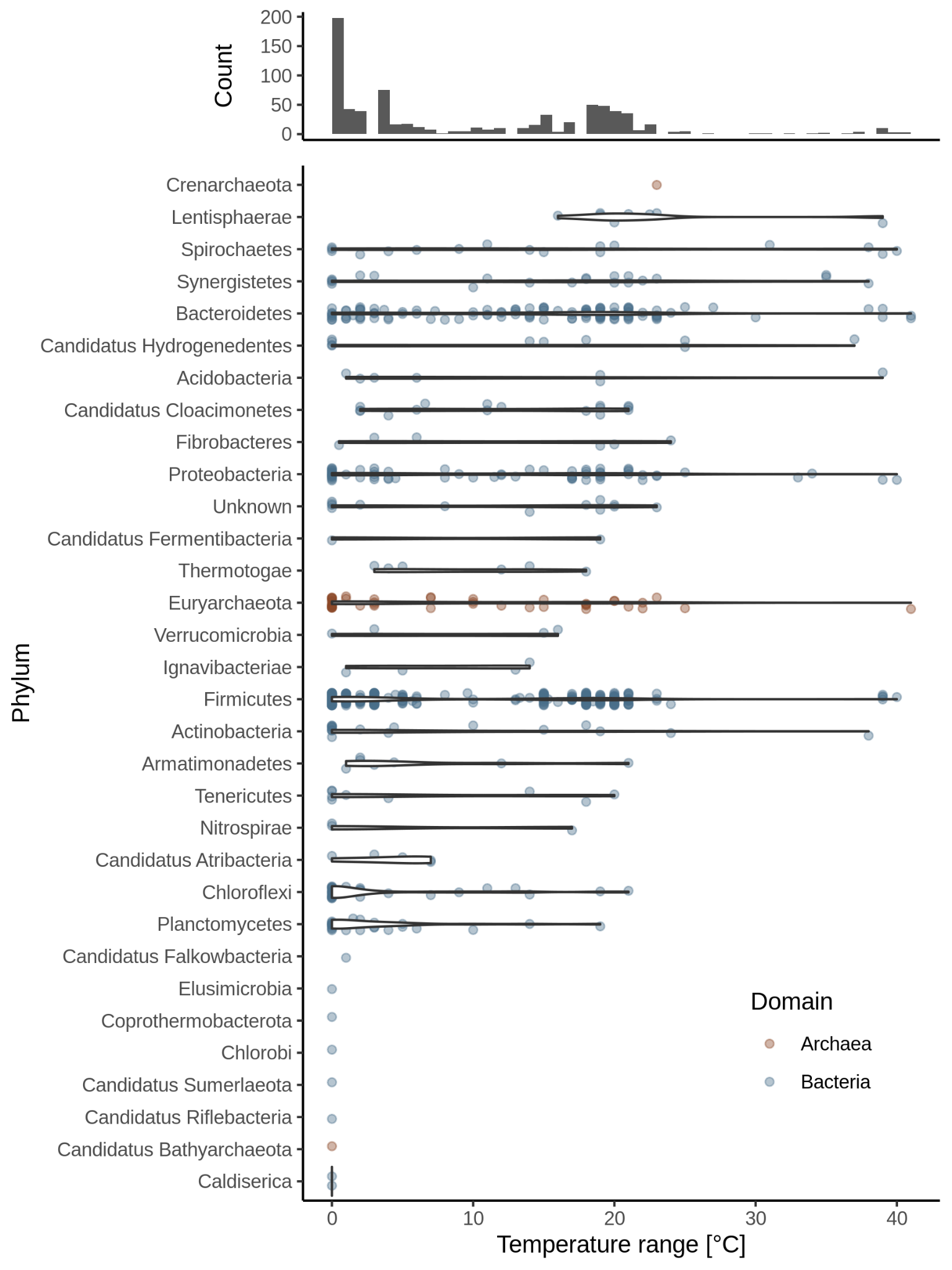


**Supplementary Figure 7. Preferential temperature range across phyla.** For each phylum, distribution of temperature ranges where MAGs have PTR values greater or equal to their median. These temperature ranges thus define the intervals where MAGs replicate faster.


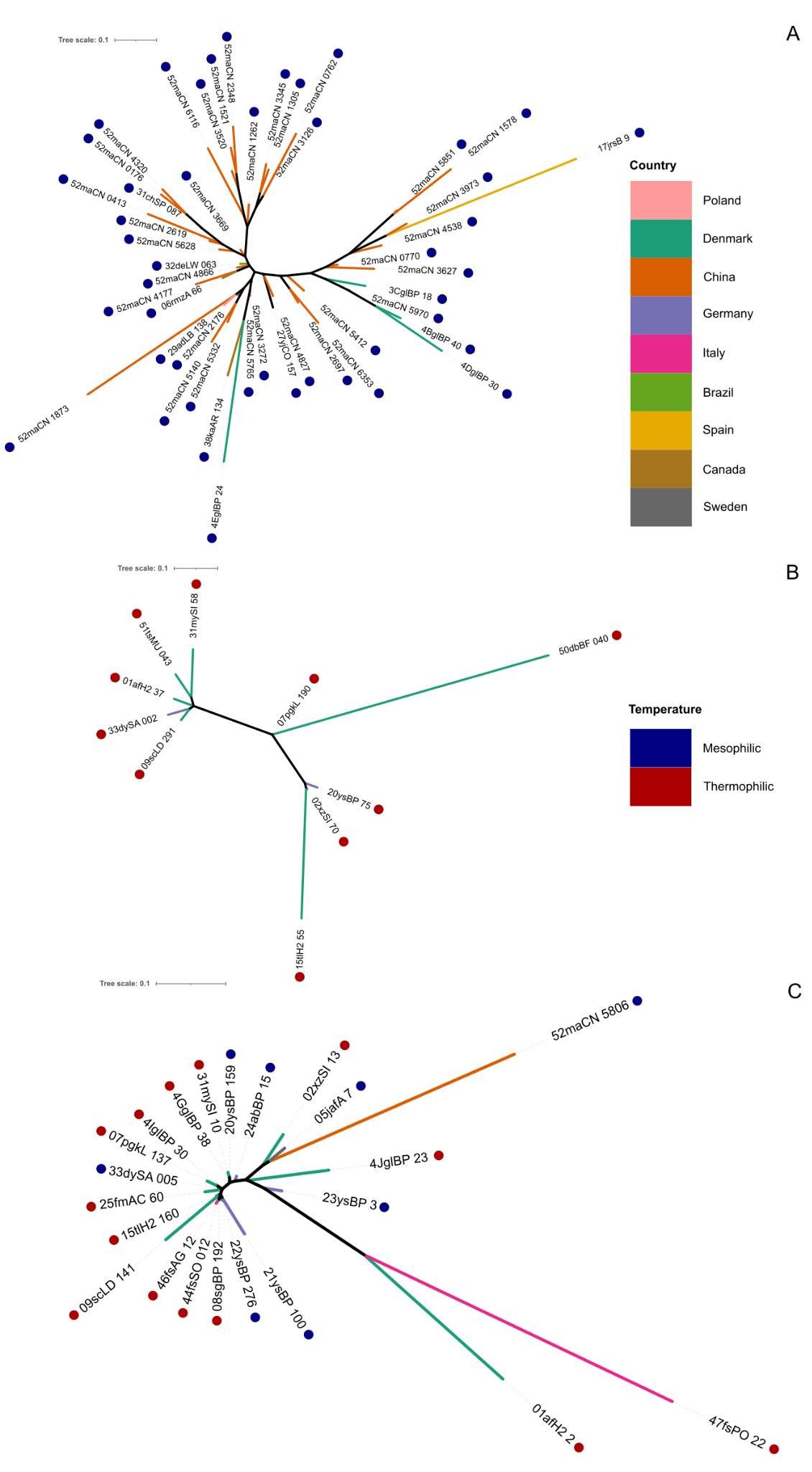


**Supplementary Figure 8. Phylogenetic analysis of the MAGs identified for three of the most common methanogens.** MAGs are associated to (A) *Methanothrix* sp. 43zhSC_152, (B) *M. wolfeii* 31mySI_58, and (C) *Ca.* M. thermohydrogenotrophicum 31mySI_10 (C). Colors associated to the branch trees are related to the country where the MAGs have been identified, while the temperature of the sample is represented by red (thermophilic) and blue (mesophilic) dots close to the MAG ID.


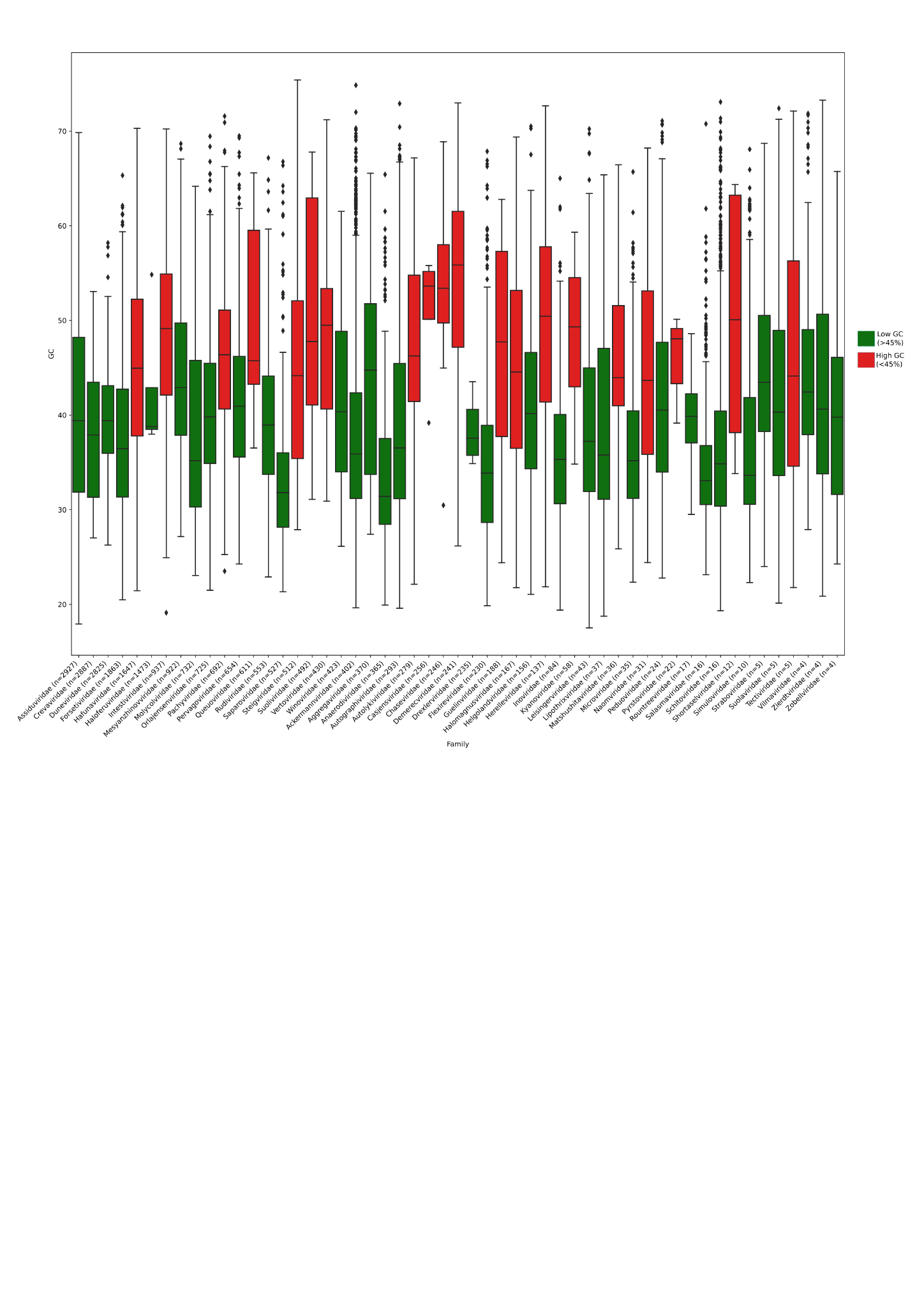


**Supplementary Figure 9. GC content distribution across phage families.** Boxplot representation of GC content distribution within phage families with at least 4 representants. Each box represents a family, with the color indicating average GC content. Families with an average GC content below 45 are shown in green, while those with an average GC content of 45 or higher are in red. The number of phages (n) in each family is also provided.
